# 10th European Conference on Rare Diseases & Orphan Products (ECRD 2020)

**DOI:** 10.1186/s13023-020-01550-1

**Published:** 2020-11-09

## Poster Presentations

### P1

#### The first effective treatment for alkaptonuria; a collaborative, patient centric effort

##### Ciarán L. Scott

###### AKU Society, Cambridge, Cambridgeshire, CB1 2BL, UK

**Correspondence:** Ciarán L. Scott - ciaran@akusociety.org

*Orphanet Journal of Rare Diseases* 2020, **15(Suppl 1)**:P1

In 1902, Sir Archibald Garrod described alkaptonuria (AKU) as inherited for the first time. Since then, there has been little or no research into a treatment for the life-changing disease that affects 1 in 250,000 people worldwide. The AKU Society was founded in 2003 to find a treatment.

In 2012 the AKU Society founded a pan-European consortium, called DevelopAKUre. This was made up of 12 members, including hospitals, pharmaceutical companies, universities, biotechs and national AKU patient groups from all over Europe who shared one aim: to prove that a repurposed drug called nitisinone works in reducing the chemical that causes the damage of AKU and that it has a positive impact on clinical features.

Together, DevelopAKUre applied for funding through the European Commission’s Seventh Framework Programme (FP7) in order to develop and run the trials that would prove the drug works. The funding secured for this programme included €6 million from the European Commission, with an additional €4 million in co-financing (for in-kind costs such as the drug supply)

Armed with this funding, we launched three studies into the efficacy and safety of the drug. The last, SONIA 2 (Suitability of Nitisinone in Alkaptonuria 2) was designed to answer once and for all if nitisinone can be used in AKU to reduce the chemical that causes the damage to bones and cartilage. The trial finished in January 2019. We found out at the end of 2019 that we had been successful. Sobi, the company that owns the rights to nitisinone, has now applied for a license for the drug’s use to treat AKU across Europe.

This is the first time an effective treatment for AKU has been found. Nitisinone has now been proven to reduce the chemical that causes AKU by up to 99%. If given early enough, the drug could prevent the disease features from developing at all.

### P2

#### ‘Patient journeys’: Personal experiences shaping clinical priorities

##### Olivia Spivack

###### European Reference Network for rare and/or complex craniofacial anomalies and ear, nose and throat disorders - Project management team, Erasmus University Medical Center, Rotterdam, The Netherlands

**Correspondence:** Olivia Spivack - o.spivack@erasmusmc.nl

*Orphanet Journal of Rare Diseases* 2020, **15(Suppl 1)**:P2

European Reference Networks (ERNs) are networks of highly specialised healthcare providers from across Europe. There are 24 networks, each focusing on a different rare disease area. ERN CRANIO is the ERN for rare and/or complex craniofacial anomalies and ear, nose and throat disorders. ERN CRANIO pools together the available disease-specific expertise, knowledge and resources from across Europe and initiates network activities designed to support patients, families and clinicians. The network also involves patient representatives in this action, who are able to engage with their patient communities and voice the needs and perspectives of patients and families.

Patient representatives within ERN CRANIO have reflected on their own personal experiences and engaged with their patient communities to develop patient journeys (PJs) [1]. PJs are disease-specific visuals, mapping identified patient/family ‘needs’ and ‘ideal support scenarios’ at key clinical stages. PJs have been developed for: Apert Syndrome, Genetic Hearing Loss, Pierre Robin Sequence, Craniofacial Microsomia, Cleft Lip/Palate and a PJ for Treacher Collins Syndrome is underway. These visuals can be continually shared, added to and developed.

The PJs can play a part in validating existing disease-specific network activities and guiding future focus. On initial inspection it also appears there is a common need for clear, easy to understand, medically accurate information on clinical diagnosis and/or treatment options. This serves to validate ERN CRANIO’s strategic focus on the development of clinical guidelines. It also highlights the importance of ensuring these guidelines are made accessible to patients and families and are communicated clearly. This may involve creating a patient version of a medical guideline, for example.

A more in-depth inspection and systematic assessment of the common needs will need to follow. However, this initial finding highlights the potential value of using the PJs to validate the network’s strategic focus and shape priorities on a broader level.

**Acknowledgements:** ERN CRANIO patient representatives have facilitated/are facilitating the completion of the patient journey content (Gareth Davies, Michel Francois, Jeroen van de Koppel, Thomas Luck, Ivana Marinac, Sandra Möshe, Elisa Nurmenniemi, Philippe Pakter, Sara Pérez and Markus Richter). ERN CRANIO is the European Reference Network for rare and/or complex craniofacial anomalies and ear, nose and throat disorders – https://ern-cranio.eu/ (FPA No. 739518).

Matt Johnson from EURORDIS Rare Disease Europe has supported the process and the creation of the visuals.

**Reference**

1. Bolz-Johnson M, Meek J, Hoogerbrugge N. “Patient Journeys”: improving care by patient involvement. *European Journal of Human Genetics.* 2019; 28 (141–143). Available from: 10.1038/s41431-019-0555-6

### P3

#### Quality of life (QoL) for people with rare diseases: Recruitment challenges and consequences in a study pilot-testing the UK-PSC-QoL, a provisional QoL tool for people with primary sclerosing cholangitis (PSC) in the UK

##### Elena Marcus^1,2^, Douglas Thorburn^2^, Patrick Stone^1^, Bella Vivat^1^

###### ^1^Marie Curie Palliative Care Research Department, Division of Psychiatry, UCL, London, UK; ^2^UCL Institute for Liver and Digestive Health, Division of Medicine, UCL & Royal Free London, NHS Foundation Trust, London, UK

**Correspondence:** Elena Marcus - elena.marcus@outlook.com

*Orphanet Journal of Rare Diseases* 2020, **15(Suppl 1)**:P3

**Aims:** We are developing a new measure of quality of life (QoL), the UK-PSC-QoL, for people with primary sclerosing cholangitis (PSC), a rare incurable disease of the bile ducts and liver which can considerably impact QoL.

**Method:** Following initial issue generation and reduction, 83 issues in six domains were constructed as items. The resulting provisional UK-PSC-QoL was then pilot-tested with PwPSC in the UK. Consensus on how to reliably stage PSC is lacking, so we hypothesised eight categories relating to disease severity, including: co-morbid inflammatory bowel disease, awaiting liver transplant, recurrent PSC, and PSC-related cancers. Participants in all categories were recruited and completed the measure. All participants with more severe PSC, plus a sub-group with less severe disease, were interviewed to explore item comprehensibility, acceptability, relevance, and redundancy. We calculated mean scores and prevalence ratios, and examined response distributions for each item.

**Results:** Sixty PwPSC completed the measure, and 25 were also interviewed. Most participants were male (57%), white British (82%), with median age 52 (range 23–75). The rarity of the condition made recruiting sufficient numbers of PwPSC challenging, particularly those more severely affected. Forty-four participants were recruited in the five less severe PSC categories, but only 16 were recruited in three categories: awaiting liver transplant (n = 5), recurrent PSC (n = 6), and with PSC-related cancer (n = 5).

Analysis of participants’ perceptions of item comprehensibility and acceptability resulted in the re-phrasing of 22 items, mostly due to item ambiguity/clarity. Six items were deleted, and one new item added. Other findings were indeterminate owing to under-recruitment in some categories.

**Conclusions:** Initial pilot-testing analysis resulted in deleting six items from the provisional UK-PSC-QoL, but recruitment challenges prevented definitive conclusions on other items. Future research, broadened internationally, would enable recruiting more people in under-represented groups, and thereby assist final decisions on item inclusion.

### P4

#### From research to practice: distal myopathy patients’ HRQoL and their need for assistance and care

##### Go Yoshizawa^1^, Shun Emoto^1^, Yuki Wakamiya^2^, Yuriko Oda^2^, Masatoshi Iwasaki^1^, Kunihiro Nishimura^1^, Soichi Ogishima^1,3^, Yukiko Nishimura^1^

###### ^1^NPO ASrid, Bunkyo Ward, Tokyo, Japan; ^2^NPO PADM, Tama City, Tokyo, Japan; ^3^Tohoku University, Sendai, Japan

**Correspondence:** Yukiko Nishimura - research@asrid.org

*Orphanet Journal of Rare Diseases* 2020, **15(Suppl 1)**:P4

**Aim:** Distal myopathy is a slowly progressive rare disease with muscle weakness starting from the limbs. Patients with distal myopathy will need assistance and care as their symptoms progress. We studied correlations between distal myopathy patients’ HRQoL (Health Related Quality of Life) and their need for assistance and care.

**Method:** The questionnaire survey was conducted in stages for Patients Association for Distal Myopathies (PADM) members via a patient online platform ‘J-RARE’. Based on open questions on their need for assistance and care, we first asked the extracted need and HRQoL (SF-12) and then asked basic attributes and activities of daily living (Barthel Index, BI; Vignos Scale, VS). As an ASrid Research Ethics Committee (REC) member and principle investigator directly exchange during the e-mail review process, REC revised the review process by setting up another mailing list for submission apart from one for review. REC then approved the study.

**Result:** Twenty-six needs extracted from 42 valid responses (male 21; age 56.0 yo; BI 40.9) at the first survey, were revised to 22 needs in consultation with PADM. Physical/mental/social QOL was 20.5/59.3/29.6 respectively on average from 28 valid responses (male 14; age 53.9 yo; BI 35.2) at the second survey. As a result of correlation analysis between 4 need-components from principal component analysis, the followings were significantly corelated (Table 1): physical QOL AND period of onset (ρ = − .06), BI (ρ = .73) and VS (ρ = − .50); mental QOL AND ‘understanding of the disease and patient’s will’ (ρ = .41); and social QOL AND ‘safe assistance and care’ (ρ = .41) and ‘smooth communication with others’ (rho = .41).

**Social implementation:** Following this study, we have issued and distributed a leaflet “Progressive muscular disease patients’ need for assistance and care” to nursing offices and other agencies across Japan since February 2020. Our approach well demonstrates how to utilize patients’ firsthand opinions in nursing practice.

**Table 1:** Correlation coefficient among HRQOL, characteristics, and needs (n = 28)

	SF-12 Subscale		
	PCS	MCS	RCS
Age	0.09	0.11	− 0.27
Duration from onset to diagnosis	0.2	0.24	− 0.27
Duration from onset to present	−**0.37**	0.31	0.12
Vignos Scale	− **0.5**	0.23	− 0.09
Barthal Index	**0.73**	− 0.28	− 0.18
Need components			
① Control and careful coordination of patient’s position	− 0.04	0.17	− 0.02
② Understanding of the disease and patient’s will and taste	− 0.05	**0.41**	− 0.2
③ Safe care	0.07	− 0.23	**0.41**
④ Smooth communication with others	− 0.11	− 0.15	**0.41**

Spearman’s ρ; PCS, Physical component summary; MCS, Mental component summary; RCS, Role/Social component summary; Bold, p < .05

**Acknowledgements:** We would like to thank the J-RARE patient organization groups and the ASrid Research Ethics Committee.

**Consent to publish:** Informed consent to publish has been obtained from patients.

### P5

#### Insights into generalized pustular psoriasis (GPP) using real-world data

##### Nirali Kotowsky^1^, Ran Gao^1^, David Singer^1^, Elizabeth M. Garry^2^, Amanda K. Golembesky^3^

###### ^1^Boehringer Ingelheim Pharmaceuticals, Inc., Ridgefield, CT, USA; ^2^Aetion, Inc., Boston, MA, USA; ^3^Boehringer Ingelheim International, GmbH, Ingelheim, Germany

**Correspondence:** Nirali Kotowsky - nirali.kotowsky@boehringer-ingelheim.com

*Orphanet Journal of Rare Diseases* 2020, **15(Suppl 1)**:P5

**Background:** Generalized pustular psoriasis (GPP) is a rare, severe, potentially life-threatening, systemic disease, characterized by recurrent acute flares consisting of disseminated erythematous skin rash with sterile neutrophil-filled pustules. By understanding the burden of disease in this population, targeted interventions that improve patient quality of life can be developed. This is the first study to use real-world data to describe healthcare resource utilization (HCRU) in patients with GPP.

**Materials and methods:** Patients were identified as having either GPP or plaque psoriasis (PsO) if they had ≥ 1 inpatient or 2 outpatient ICD-10 (International Classification of Disease, tenth revision) diagnosis codes (L40.1 or L40.0, respectively), separated by 30–365 days. All analyses were conducted via the Aetion Evidence Platform^®^ v3.17, using Optum^®^ Clinformatics™ Data Mart, a US administrative claims database. The study period was from October 1, 2015 to March 31, 2019, with the first diagnosis code marking the index date. A general population matched cohort (MC) of 4:1, based on age and sex, was generated for context. No formal comparisons were conducted. Patient characteristics, treatment and all-cause HCRU calculated for each visit type (inpatient, outpatient and emergency department [ED]) during the 12-month follow-up (FU) were analyzed.

**Results:** In total, 1669 patients with GPP and 60,419 patients with PsO were identified. Of these, 1014 patients with GPP and 32,665 patients with PsO had ≥ 12-month FU. During baseline, patients with GPP vs PsO and the MC were more likely to suffer from psoriatic arthritis (GPP: 11.8%; PsO: 7.0%; MC: 0.1%), anxiety (GPP: 11.6%; PsO: 6.7%; MC: 5.8%) and depression (GPP: 10.7%; PsO: 5.5%: MC: 5.0%). During the 12-month FU, patients with GPP vs PsO and the MC had more outpatient visits (mean number of visits, GPP: 28.7; PsO: 22.6; MC: 15.3), a higher proportion of patients with GPP had inpatient admissions (GPP: 22.5%; PsO: 9.4%; MC: 10.4%) and ED visits (GPP: 40.5%; PsO: 22.3%; MC: 23.0%), and were hospitalized for longer (mean duration, GPP: 16.8 days; PsO: 11.8 days; MC: 14.4 days).

**Conclusions:** This analysis suggests that patients with GPP have a greater burden of comorbidities vs those with PsO. It also suggests that patients with GPP have greater HCRU vs PsO patients or the MC. These results highlight an unmet clinical need among patients with GPP.

**Acknowledgements:** Editorial support was provided by Amy Pashler, PhD, from OPEN Health Medical Communications (London, UK) and was funded by Boehringer Ingelheim.

### P6

#### Meeting challenges in evaluating and measuring functioning in rare diseases: a collaboration between Orphanet and Mapi Research Trust

##### Annie Olry^1^, Gavin McDonough^1^, Charlotte Rodwell^1^, Ana Rath^1^, Benoit Arnould^2^, Celine Desvignes-Gleizes^2^, Laure-Lou Perrier^2^, Catherine Acquadro^2^

###### ^1^INSERM, US14 – Orphanet, Plateforme Maladies Rares, Paris, France; ^2^Mapi Research Trust, Lyon, France

**Correspondence:** Annie Olry - annie.olry@inserm.fr

*Orphanet Journal of Rare Diseases* 2020, **15(Suppl 1)**:P6

**Theme:** When therapies meet the needs: enabling a patient-centric approach to therapeutic development.

**Background:** Rare diseases (RD) often result in a wide spectrum of disabilities, on which information is lacking. There is a need for standardised, curated data on the functional impact of RD to facilitate the identification of relevant Patient Reported/Patient Centered Outcome Measures (PROMs/PCOMs) as well as for the use of validated Quality of Life instruments based on functional outcomes. To address these issues, Orphanet is partnering with Mapi Research Trust (MRT) in order to connect Orphanet to PROQOLID™, MRT’s PROMs/PCOMs database, through disease codes. Visit Orphanet at www.orpha.net.

**Methods and materials**: The Orphanet Functioning Thesaurus (OFT) is a multilingual controlled vocabulary derived from the ICF-CY. A subset of RD present in the Orphanet nomenclature is annotated with the OFT, with the addition of attributes for each functional impact (frequency, severity, and temporality) for each specific RD. Annotations result from structured interviews with clinical experts, medical-social sector care providers, and patient organisations.

In order to link PROQOLID™ data with Orphanet disability data, the taxonomy used to qualify RDs in PROQOLID™ was reviewed and mapped to Orphanet’s. All PROMs developed for RDs were identified, and all products approved by the FDA and EMA from 2002 to 2017 with an orphan drug designation (ODD) and a PRO claim were listed.

**Results:** The Orphanet knowledge base contains over 6000 RD, of which 1073 RD have been assessed for their functional consequences, of which 675 RD have been annotated: the remaining 398 RD were annotated, after discussions with medical experts, as either being highly variable, non-applicable or resulting in early-death. Of the 390 most prevalent rare diseases, 156 have been annotated according to their functional consequences. 64 RDs had a PROM (n = 144) in PROQOLID™ and 17.4% of ODD included a PRO claim. The RDs with the most PROM were sickle cell anemia, spinal cord injuries, cystic fibrosis, all forms of hemophilia A and B and Duchenne Muscular Dystrophy. PROM used in labels were primarily focusing on symptoms (100%), rarely on functioning (4%) or health-related quality of life (12%).

Conclusions: Linking these two databases, and providing standardised, curated data, will enable the community to identify PROMs/PCOMs for RD, and is the first step towards validated Quality of Life instruments based on functional outcomes.

### P7

#### Patients’ view on the unmet need in endocrine medical research

##### Johan de Graaf^1^, Friso de Vries^2^, Anne Dirkson^3^, Olaf Hiort^4^, Alberto M. Pereira^2,5^, Marta Korbonits^6^, Martine Cools^7^

###### ^1^ePAG of WP Research and Science and Chair of Dutch Pituitary Foundation, Nijkerk, The Netherlands; ^2^Department of Medicine, Division of Endocrinology and Centre for Endocrine Tumors Leiden (CETL), Leiden University Medical Centre, Leiden, the Netherlands; ^3^Leiden Institute for Advanced Computer Science, Leiden University, Leiden, the Netherlands; ^4^Paediatric Chair and deputy coordinator of Endo-ERN, Division of Paediatric Endocrinology and Diabetes, Department of Paediatrics and Adolescent Medicine, University of Lübeck, Lübeck, Germany; ^5^Adult Chair and coordinator of Endo-ERN, Leiden, The Netherlands; ^6^Adult chair of WP Research and Science and Centre for Endocrinology, William Harvey Research Institute, Barts and the London School of Medicine and Dentistry, Queen Mary University of London, UK; ^7^Paediatric chair of WP Research and Science and Department of Paediatric Endocrinology, Ghent University Hospital, University of Ghent, Ghent, Belgium

**Correspondence:** Johan de Graaf - j.degraaf@hypofyse.nl

*Orphanet Journal of Rare Diseases* 2020, **15(Suppl 1)**:P7

**Background:** The mission of the European Reference Network on Rare Endocrine Conditions (Endo-ERN) is to reduce and ultimately abolish inequalities in care for patients with rare endocrine conditions in Europe through facilitating knowledge sharing and facilitating related healthcare and research. Endo-ERN consists of > 80 expert centres from 26 European countries and ensures equality between paediatric and adult patient involvement through 15 European Patient Advocacy Group representatives co-chairing the Steering Committee. In order to incorporate the patients perspective on medical research this study aims to assess which topics rare endocrine disease patients see as a priority for medical research.

**Materials and methods:** A survey was developed, translated in 22 different European languages, and distributed to European patients with rare endocrine conditions with the aid of the networks of Endo-ERN and Eurordis. Some patient organisations distributed the survey too more common disease patients as well, e.g. Hashimoto’s disease, and these responses were excluded. The survey asked patients to give suggested topics (i.e. fertility, heritability, tiredness, daily medicine intake, sleep quality, physical discomfort, and ability to work, partake in social life, and sports) a priority score and to suggest their own topics for research in open fields. Open field responses were analysed with topic modelling and KLIPP-analysis.

**Results:** After exclusion of responses from more common endocrine disease patients, 1378 survey responses were analysed. Most responses were received from Northern (47%) and Western Europeans (39%), while Southern (11%) and Eastern Europe (2%) were underrepresented. Of the suggested topics respondent were most interested in research concerning the ability to work and participate in social life, and on tiredness. When patients were open to suggest their own topics, common responses included long-term side effects of drugs and quality of life. However, priorities differed between disease groups. For example, adrenal, pituitary and thyroid patients were more interested in research concerning tiredness than others.

**Conclusion:** With this survey Endo-ERN is provided with a large sample of responses from European patients with a rare endocrine condition, and those patients experience unmet needs in research, though these needs differ between the disease groups. The results of this study should be incorporated by clinical experts in the design of future studies in the rare endocrine disease field.

### P8

#### The Cystic Fibrosis Community Advisory Board (CF CAB) – How we measure our successes

##### Marja J. Nell^1^, Rob Camp^2^, Hilde De Keyser^3^

###### ^1^CAB, Nederlandse Cystic Fibrosis Stichting, Baarn, The Netherlands; ^2^CAB, EURORDIS, Paris, France; ^3^Cystic Fibrosis Europe, Brussels, Belgium

**Correspondence:** Hilde De Keyser - hilde.dekeyser@cf-europe.eu

*Orphanet Journal of Rare Diseases* 2020, **15(Suppl 1)**:P8

**Background:** The Cystic Fibrosis (CF) Community Advisory Board (CAB) is an independent pan-European group of trained patients and patient representatives from 13 European countries, who meet on a regular basis with representatives of CF companies and other CF stakeholders involved in research, development and treatment access discussions. The CF CAB was established in 2017, their mission being to ensure that the patient perspective is always considered and incorporated, throughout all stages of research and treatment development.

**Methods:** Meetings with CF companies are followed up with letters and a questionnaire. With each company, a “tracker” is established to maintain the interaction and measure success. This company tracker is an overarching reference document, used to set goals, capture outcomes and measure progress and success for each company. To evaluate the effectiveness of the CAB, a survey is sent to both the companies that have participated in CAB meetings and the CAB members.

**Results:** The CF CAB has held 6 meetings with 5 different companies in the past three years. All companies responded to the survey. The CAB meetings were experienced as extremely to very useful. Main outcomes of the meetings were:

All companies responded that they will continue their relationship with the CAB; companies identified distinct patient-relevant outcomes; all companies answered that CAB meetings helped them to demonstrate the value of their product to HTA agencies and to regulators; furthermore, the meeting helps to some degree in making clinical study programmes more aligned with patients’ needs; based on the input from the CAB more than half reviewed specific parts of studies or research plans. The tracker is seen as a useful instrument for future collaboration.

**Conclusion:** Companies highly valued the input from the CF CAB. The most important criteria for a successful liaison were good follow-up and maintain the tracker active.

### P9

#### The Value of Patient Engagement in Research Design: The EURORDIS patient-led Community Advisory Boards (CABs)

##### Rob Camp, François Houÿez

###### EURORDIS, Paris, France 75014

**Correspondence:** Rob Camp - rob.camp@eurordis.org

*Orphanet Journal of Rare Diseases* 2020, **15(Suppl 1)**:P9

**Purpose**: When developing a health technology that requires clinical studies, developers institute working relations with clinical investigators. Patient representatives can also create and manage advisory boards with product developers. This was of high utility in the 1990s, in the development of products to treat HIV infection. Inspired by this model, the European Organisation for Rare Diseases (EURORDIS) proposes the EuroCAB programme to facilitate a two-way dialogue between patient representatives and medicine developers. As of 2019, 6 disease-specific CABs exist of approximately 12 members each and others are being formed.

**Methods**: EURORDIS invites developers to sign a Charter for collaboration with patients in clinical research, and provides guidelines together with a mentoring and training programme for patient networks. CABs help set the agenda with the developer, work on topics as diverse as study design, feasibility, informed consent and site selection, QoL and PROMs, and organize the meetings. Discussions also cover compassionate use, pricing, relative efficacy, etc. Meetings last for 2 to 4 days with sessions with different developers, all under confidentiality. There are regular between-meeting teleconferences for trainings and action plan updates, and some CABs have instituted working groups on access, psychological support, etc. The collaboration is evaluated via a post-meeting survey send to both CAB members and medicine developers. In addition, CABs have recently started to monitor outcomes of the meeting and progress towards their goals with a tracker tool.

**Results**: The results of the first surveys from 14 distinct CAB meetings with 19 companies show that this form of shared decision-making is valuable as well as ethical for both parties. We have seen that working relations always continue, even when discussions become heated. All involved show interest in the co-creation possibilities of such collaboration and we look forward to seeing progress and change via the tracker.

**Conclusions**: Monitoring and evaluation are crucial to understand whether and how the CABs are making an impact on medicine development. Demonstrating impact is challenging because of the contextualized nature and complexity inherent to patient engagement collaborations in research design. EURORDIS is working within PARADIGM on our monitoring and evaluation strategy, focusing on improving its comprehensiveness and including multi-stakeholder perspectives. Our current experiences show that the EuroCAB programme, with collective thinking and exchange between patients and a collaborative mentality from both sides, ensures high-quality and constructive dialogue with researchers and developers and can eventually inform both HTA and regulatory decision-making (Fig. 1). We have started to work on the metrics of markers of success.

**Figure:**
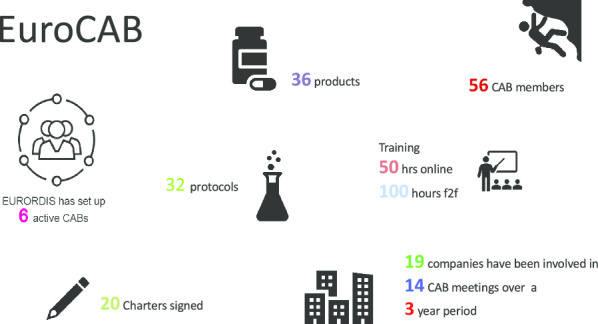
**Fig. 1** Graphic of work produced by the CABs in the EUROCAB programme over the last 3 years

**Acknowledgements:** Special thanks for thoughtful input to our PARADIGM partners at VU-Athena, Amsterdam, NL.

### P10

#### How to welcome visually impaired people to the hospital? Educational video for healthcare professionals

##### Caroline Iberg^1,2^, Marilyne Oswald^2^, Claire Edel^1,2^, Dorothée Leroux^1^, Fédération des Aveugles Alsace Lorraine Grand Est^3^, Fédération des Aveugles de France^4^, Hélène Dollfus^1,2,^^5^

###### ^1^ERN-EYE Coordination Team, Hôpitaux Universitaires de Strasbourg, Strasbourg, France; ^2^SENSGENE Coordination Team, Hôpitaux Universitaires de Strasbourg, Strasbourg, France; ^3^ www.aveugles-grand-est.fr, Strasbourg, France; ^4^ www.aveuglesdefrance.org, Paris, France; ^5^CARGO, Hôpitaux Universitaires de Strasbourg, Strasbourg, France

**Correspondence:** Caroline Iberg - caroline.iberg@chru-strasbourg.fr

*Orphanet Journal of Rare Diseases* 2020, **15(Suppl 1)**:P10

**Purpose:** The French national Network for rare sensory diseases SENSGENE launched in 2019 a 3-min motion design video (Fig. 1) aiming at guiding healthcare professionals to welcome visually impaired patients in the hospital. This educational video was created to address patients’ expectations and improve their experience in the network’s hospital. The European Reference Network for Rare Eye Diseases (ERN-EYE) collaborated on the project and created an English version of the video in order to distribute it widely in Europe.

**Figure:**
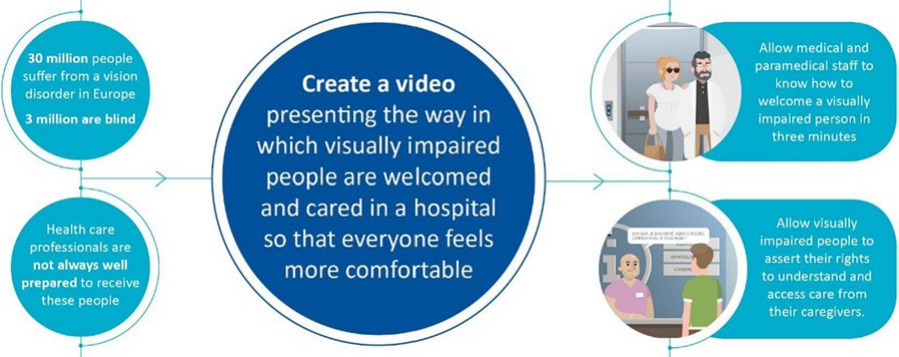
**Fig. 1** Strategic development of the video: issues and objectives

**Method:** SENSGENE worked on this project with two big French associations of visually impaired people: Fédération des Aveugles et Amblyopes de France and Fédération des Aveugles Alsace Lorraine Grand Est. More than 15 French patients’ associations actively contributed to the project through five focus groups (workshops) which collected testimonies and gathered the needs of visually impaired persons and health-care professionals (Fig. 2). An evaluation was made by the independent body IPSO FACTO: 30 health professionals answered to a survey before and after viewing the video.

**Figure:**
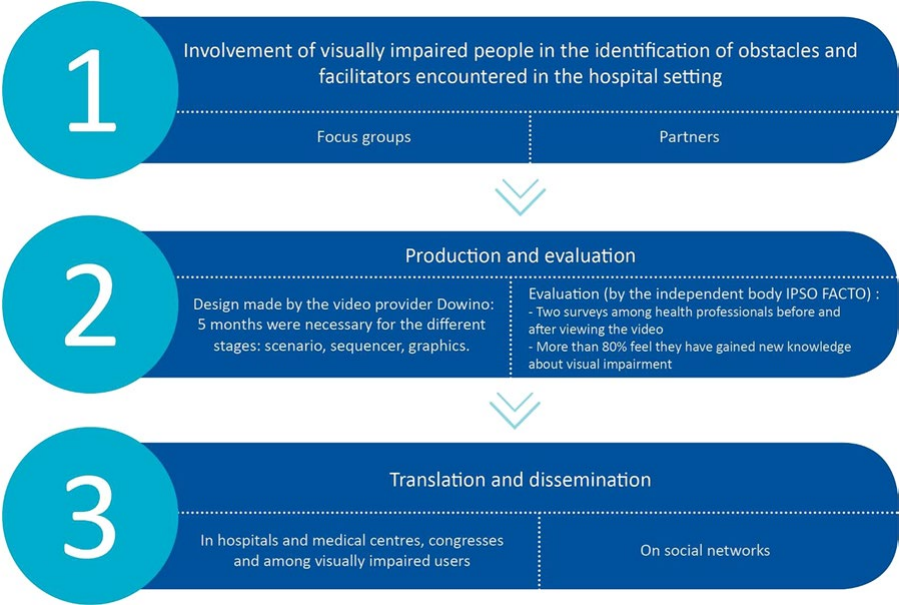
**Fig. 2** The project approach was structured around several steps

**Results:** The video deals with common situations in the delivery of care activities for different types of visual impairment: reception in a hospital center, consultations, moves and orientation in a hospital room (Fig. 3). This fits perfectly with the needs of the patients reported in the focus groups. Besides, the evaluation showed that 80% of them improved their knowledge on the topic.

**Figure:**
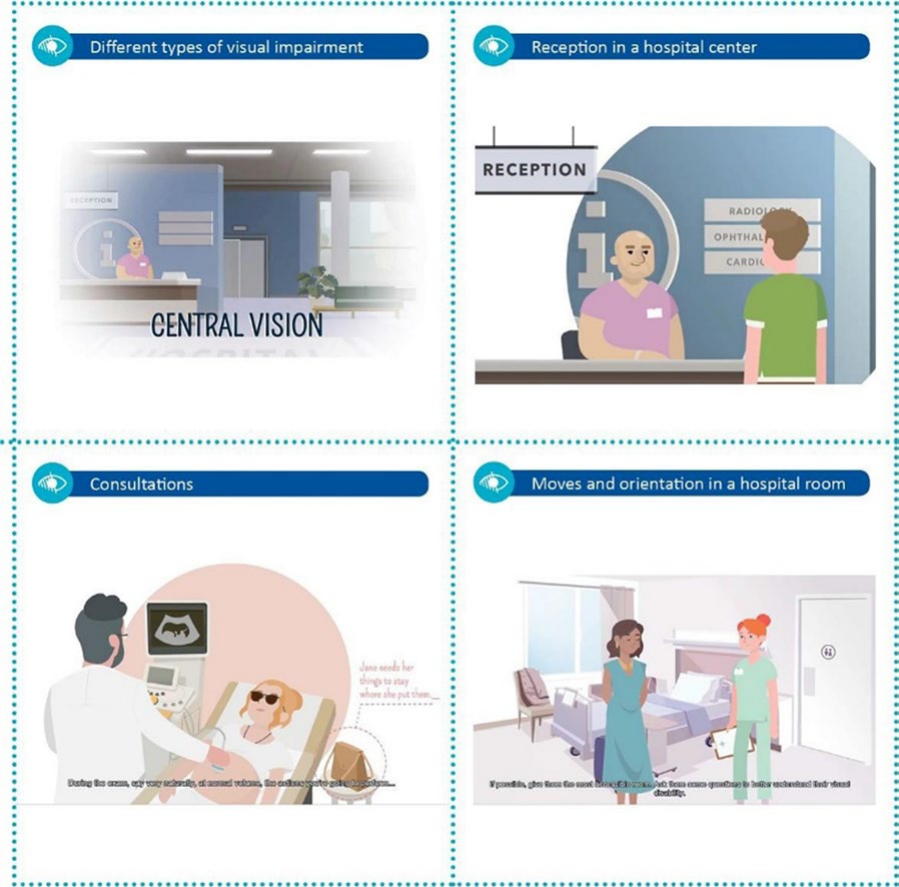
**Fig. 3** The different sequences of the video

**Figure:**
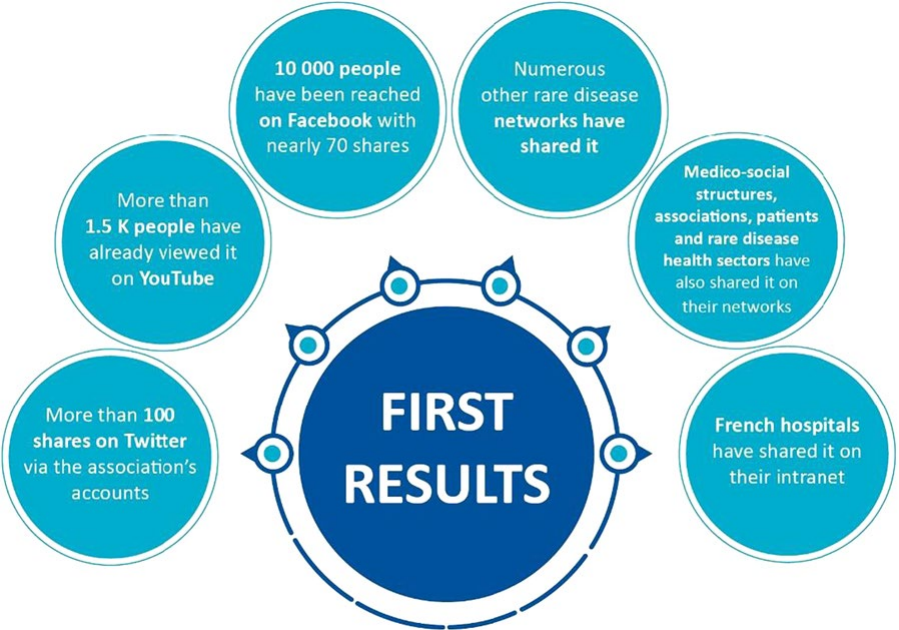
**Fig. 4** First results after 6 months of dissemination

**Conclusion:** This video was a great success in France since its launch in October 2019 (Fig. 4). Thus, as it was created thanks to the involvement of patients and healthcare professionals, it fits a real need. Thanks to the English translation and spreading through the ERN-EYE network and international congresses, it is now widely distributed all over Europe.

To watch the video: https://www.youtube.com/watch?v=sHniVmgKung&t=1s.

**Acknowledgements:** This project received the financial support of SENSGENE [http://www.sensgene.com/], the Caisse nationale de Solidarité pour l’Autonomie - National Solidarity and Autonomy Fund [https://www.cnsa.fr/], and ERN-EYE [https://www.ern-eye.eu]. ERN-EYE is partly co-funded by the European Union within the framework of the Third Health Programme “ERN-2016 - Framework Partnership Agreement 2017-2021 No 739534”.

This video was made in partnership with the Blind Federation of France (Fédération des Aveugles et Amblyopes de France) [htttps://www.aveuglesdefrance.org], as well as with the Blind Federation of Alsace Lorraine Grand Est [https://www.aveugles-grand-est.fr/].

Member associations of SENSGENE participated in the focus groups: Association Bardet-Biedl [https://www.bardet-biedl.com/], Association contre les Maladies Mitochondriales [http://www.association-ammi.org/], Association France Choroïdéremie [https://france-choroideremie.org/], Association Genespoir [http://www.genespoir.org/], Association Gêniris [https://www.geniris.fr/], Association Inflam’œil [http://www.inflamoeil.org/], Association KJER France [https://www.kjer-france.org/], Association Microphtalmie France [http://asso-microphtalmie.org/blog/], Association Mouvement Nystagmus [http://www.nystagmus.fr/], Association Ouvrir les yeux [http://www.ouvrirlesyeux.org/], Retina France [http://www.retina.fr/], Association Française Sturge-Weber “Vanille-Fraise” [http://www.vanille-fraise.org/], Association Syndrome de Wolfram [https://association-du-syndrome-de-wolfram.org/], UNAPEDA [http://www.unapeda.asso.fr/], Association Valentin Haüy [https://www.avh.asso.fr/fr], Association Vision’Ere [http://www.facebook.com/pg/association.visionere68/about/?ref=page_internal].

The design was made by the provider Dowino creation studio [https://www.dowino.com/] in Lyon, France. They allowed the ideas stemming from the focus groups to take shape and life under the pen of their graphic designer.

The evaluation was carried out by the Blind Federation of France with the independent body IPSO FACTO Institute [http://ipsofacto-co.fr/].

Special thanks to Russell Wheeler, English European Patient Advocacy Group (ePAG) of ERN-EYE, who helped us with the translation

### P11

#### The Impact of Country Specific Methods of Appraising Rare Disease Treatments

##### Amanda Whittal^1^, Elena Nicod^1^, Sheela Upadhyaya^2^, Mike Drummond^3^, Karen Facey^4^

###### ^1^SDA Bocconi School of Management, Milan, Italy; ^2^NICE, London, United Kingdom; ^3^University of York, York, United Kingdom; ^4^University of Edinburgh, Edinburgh, United Kingdom

**Correspondence:** Elena Nicod

*Orphanet Journal of Rare Diseases* 2020, **15(Suppl 1)**:P11

**Background:** Traditional appraisal and reimbursement approaches such as cost/QALY are increasingly recognised as being potentially unsuitable for rare disease treatments (RDTs). Approaches to appraising RDTs vary across countries, from the same processes used for all medicines, to those completely separate from the standard, to adapted standard processes with greater willingness to pay (WTP). This study examines the impacts of standard versus special appraisal processes for specific RDTs in selected countries.

**Methodology:** A case study analysis was conducted in which countries with a variety of RDT appraisal processes were selected, along with two RDTs representative of the following criteria: rare/ultra-rare treatment, affecting child/adult, cancer/non-cancer, life-threatening/disabling. Public HTA reports for each country’s appraisal of the selected RDTs were retrieved and used to extract information into pre-designed templates, which allowed for systematic comparison of the RDT processes across countries to compare and exemplify the impact of the different processes.

**Results:** Reports from Belgium, England, France, Germany, U.S., Italy, Lithuania, Netherlands, Poland, Romania, Scotland, Slovakia, and Sweden were selected for Spinraza and Voretigene. Characteristics of each country’s process were extracted, including special reimbursement for RDTs, special RDT committees, economic evaluation modifications, greater WTP, quality of evidence flexibility, additional considerations, etc. Special and standard processes seemed to have different impacts on the appraisal of RDTs. Special processes more consistently managed RDT issues such as evidential uncertainty and higher ICERs. Standard processes sometimes informally applied some of the characteristics included in special processes, such as broader consideration of value.

**Conclusions:** Comparing case study country examples of RDT appraisal exemplified the complexity of these processes. Special processes were more consistent in managing the challenges in RDT appraisal than standard processes.

**Practical application:** Findings suggest a need for adapted approaches for RDT appraisal, to facilitate management of associated challenges and more consistent decision-making.

### P12

#### Estimating the broader fiscal impact of rare diseases using a public economic framework: A case study applied to acute hepatic porphyria (AHP)

##### Mark P. Connolly^1,2^, Nikos Kotsopoulos^1,3^, Julien Patris^4^

###### ^1^Global Market Access Solutions Sarl, St-Prex, 1162 Switzerland; ^2^Unit of Pharmacoepidemiology & Pharmacoeconomics, Department of Pharmacy, University of Groningen, Groningen, The Netherlands; ^3^University of Athens, Department of Economics, Athens, Greece; ^4^Alnylam Pharmaceuticals, Amsterdam, The Netherlands

**Correspondence:** Mark P. Connolly - mark@gmasoln.com

*Orphanet Journal of Rare Diseases* 2020, **15(Suppl 1)**:P12

**Background:** The aim of this study was to apply a public economic framework to evaluate a rare disease, acute hepatic porphyria (AHP) taking into consideration a broad range of costs that are relevant to government in relation to social benefit payments and taxes paid by people with AHP. AHP is characterized by potentially life-threatening attacks and for many patients, chronic debilitating symptoms that negatively impact daily functioning and quality of life. The symptoms of AHP prevent many individuals from working and achieving lifetime work averages. We model the fiscal consequences for government based on reduced lifetime taxes paid and benefits payments for a person diagnosed aged 25 experiencing 12 attacks per year.

**Materials & Methods:** A public economic framework was developed exploring lifetime costs for government attributed to an individual with AHP in Sweden. Work-activity and lifetime direct taxes paid, indirect consumption taxes and requirements for public benefits were estimated based on established clinical pathways for AHP and compared to the general population (GP).

**Results:** Lifetime earnings are reduced in an individual with AHP by SEK6.5 million compared to the GP. This also translates to reduced lifetime taxes paid of SEK2.8 million for an AHP individual compared to the GP. We estimate increased lifetime disability benefits support of SEK3.1 million for an AHP individual compared to GP. We estimate average lifetime healthcare costs for AHP individual of SEK31.9 million compared to GP of SEK2.5 million. These estimates do not include other societal costs such as impact on caregiver costs.

**Conclusions:** Due to severe disability during the period of constant attacks, public costs from disability are significant in the AHP patient. Lifetime taxes paid are reduced as these attacks occur during peak earning and working years. The cross-sectorial public economic analysis is useful for illustrating the broader government consequences attributed to health conditions.

**Ethics Approval:** The study results described here are based on a modeling study. No data on human subjects has been collected in relation to this research.

### P13

#### The European Cystic Fibrosis Society Patient Registry’s Data Quality programme

##### Jacqui van Rens^1^, Alice Fox^2^, Marko Krasnyk^2^, Annalisa Orenti^3^, Anna Zolin^3^, Lutz Naehrlich^4^, on behalf of the European Cystic Fibrosis Society Patient Registry

^1^University Hospital Leuven, Leuven, Belgium; ^2^European Cystic Fibrosis Society Patient Registry, Karup, Denmark; ^3^Department of Clinical Sciences and Community Health, Laboratory of Medical Statistics, Epidemiology and Biometry G. A. Maccacaro, University of Milan, Milan, Italy; ^4^Department of Pediatrics, Justus-Liebig-University Giessen, Giessen, Germany

**Correspondence:** Jacqui van Rens - ecfs-pr@uzleuven.be

*Orphanet Journal of Rare Diseases* 2020, **15(Suppl 1)**:P13

**Background:** The European Cystic Fibrosis (CF) Society Patient Registry collects demographic and clinical data from consenting people with CF in Europe. The Registry’s database contains data of over 49,000 patients from 38 countries. High quality data is essential for use in annual reports, epidemiological research and postauthorisation studies.

**Methods:** A validation programme was introduced to quantify consistency and accuracy of data-input at source level and verify that the informed consent, required to include data in the Registry, has been obtained in accordance with local and European legislation. Accuracy is defined as the proportion of values in the software matching the medical record, consistency as definitions used by the centre matching those defined and required by the Registry. Data fields to verify: demographic, diagnostic, transplantation, anthropometric and lung function measurement, bacterial infections, medications and complications. The number of countries to validate: 20% of the total countries/year. In the selected country ≥ 10% of the centres should be visited and 15–20% of the data validated.

**Results:** In 2018, ten out of 41 centres (24%) in 4 countries (Austria, Portugal, Slovakia, Switzerland) with ≥ 50% of all patients in their countries were selected. In a 1 day visit, the data of the Registry were compared with the medical records, the outcomes and recommendations discussed, and a final report provided. Demographic, diagnostic and transplant data were checked for 489 patients (21%*), clinical data for 463 patients (20%*) (2016 data). Challenges were: informed consent, mutation information (genetic laboratory report missing), definitions interpretations. See Fig. 1 for the results.

**Figure:**
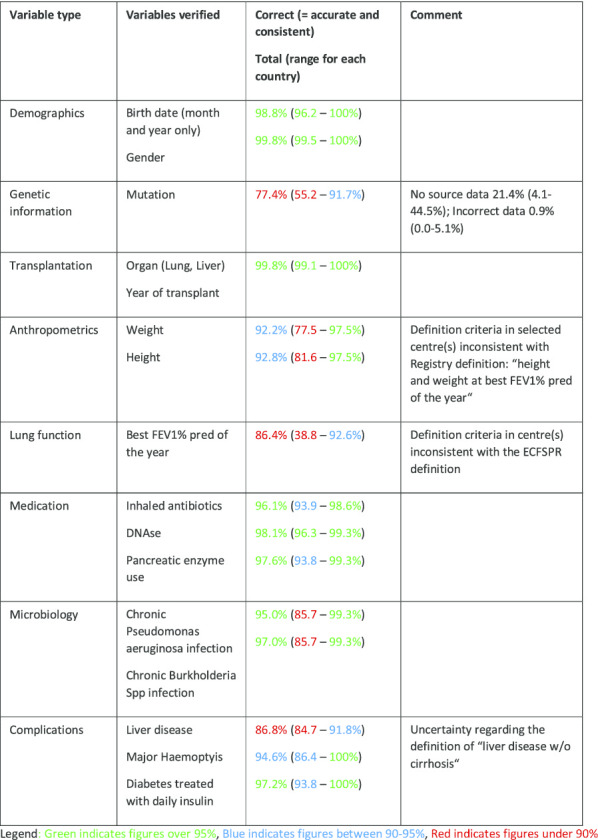
**Fig. 1** Green indicates figures over 95%, blue indicates figures between 90 and 95%, red indicates figures under 90%

**Conclusion:** The Registry’s data is highly accurate for most data verified. The validation visits proved to be essential to optimise data quality at source, raise awareness of the importance of correct informed consent and encourage dialogue to gain insight in how procedures, software, and support can be improved.

*Of the total patients in these countries.

**Acknowledgements:** We thank the following centres for participating in this project: Austria: Sabine Renner and Brigitte Mersi (Universitätsklinik für Kinder und Jugendheilkunde, Wien), Helmut Ellemunter and Johannes Eder (Medizinische Universität Innsbruck), Andreas Pfleger (Medizinische Universität Graz). Portugal: Luisa Pereira and Pilar Azevedo (Hospital Santa Maria, Centro Hospitalar Lisboa Norte), Fernanda Gamboa and Teresa Silva (Hospitais da Universidade de Coimbra). Slovakia: Hana Kayserova and Nina Bliznakova (Klinika detskej pneumologie, SZU UN Bratislava). Switzerland: Andreas Jung (Kinderspital Zürich), Philip Latzin and Romy Rodriguez (Inselspital Bern), Christian Benden and Thomas Kurowski (Universitätsspital Zürich), Reta Fischer and Patrizia Bevilacqua (Quartier Bleu, Bern).

### P14

#### Evidence of content validity of the Duchenne Video Assessment scorecards from a Delphi panel study

##### Marielle G. Contesse^1^, Linda Lowes^2^, Michelle K. White^3^, Laura Dalle Pazze^4^, Christine McSherry^1^, Mindy Leffler^1^

###### ^1^Casimir, Plymouth, MA, USA; ^2^Nationwide Children’s Hospital, Columbus, OH, USA; ^3^Optum, Johnston, RI, USA; ^4^Charley’s Fund, New York, NY, USA

**Correspondence:** Marielle G. Contesse - mariellec@casimirtrials.com

*Orphanet Journal of Rare Diseases* 2020, **15(Suppl 1)**:P14

**Background:** People with Duchenne muscular dystrophy (DMD) adopt compensatory movement patterns to maintain independence as muscles get weaker. The Duchenne Video Assessment (DVA) tool provides a standardized way to document and assess quality of movement. Caregivers video record patients doing specific movement tasks at home using a secure mobile application. Physical therapists (PTs) score the videos using scorecards with prespecified compensatory movement criteria.

**Objective:** To gather expert input on compensatory criteria indicative of clinically meaningful change in disease to include in scorecards for 15 movement tasks.

**Approach:** We conducted 2 rounds of a Delphi panel, a method for building consensus among experts. We recruited 8 PTs who have evaluated ≥ 50 DMD patients in clinic and participated in ≥ 10 DMD clinical trials. In Round 1, PTs completed a preliminary questionnaire to evaluate compensatory criteria clarity and rate videos of 4 DMD patients performing each movement task using scorecards. In Round 2, PTs participated in an in-person discussion to reach consensus (≥ 75% agreement) on all compensatory criteria with disagreement or scoring discrepancies during Round 1.

**Results:** Of the 8 PTs, 38% practiced physical therapy for ≥ 20 years, 75% provided physical therapy to ≥ 200 DMD patients, and 38% participated in ≥ 15 DMD clinical trials. Of 153 version 1 compensatory criteria, 70 (46%) were revised in Round 1. Of 150 version 2 compensatory criteria, 97 (65%) were revised in Round 2. The 8 PTs reached 100% agreement on all changes made to scorecards during the in-person discussion except the Run scorecard due to time restrictions. A subset of the panel (3 PTs) met after the in-person discussion and reached consensus on compensatory criteria to include in the Run scorecard.

**Conclusion:** Expert DMD PTs confirmed that the compensatory criteria included in the DVA scorecards were appropriate and indicative of clinically meaningful change in the disease.

### P15

#### Can wearable sensor technology support a paradigm shift in paediatric rare disease research?

##### Cécile Ollivier^1^, Phillip Griffiths^2^, Dr Aimee Donald^3^, Anna Mayhew^4^, Elin Haf Davies^1^

###### ^1^Business Development, Aparito, Wrexham, Wrexham, LL13 7YP, UK; ^2^Psychometrics, Adelphi Values, Bollington, Cheshire, SK10 5JB, UK; ^3^Metabolic Department, Royal Manchester Children’s Hospital, Manchester, Greater Manchester, M13 9WL, UK; ^4^John Walton Muscular Dystrophy Research Centre, Great North Children’s Hospital, Newcastle upon Tyne, Tyne and Wear, NE1 4LP, UK

**Correspondence:** Cécile Ollivier

*Orphanet Journal of Rare Diseases* 2020, **15(Suppl 1)**:P15

**Introduction:** Fifty percent of rare disease cases occur in childhood. Despite this significant proportion of incidence, only 17% of adult medicines authorised by the European Medicines Agency (EMA) completed paediatric trials [1,2]. As a result, many clinical needs are left unmet.

Various factors compound the development of treatments for paediatric rare diseases, including the need for new Clinical Outcome Assessments (COAs), as conventional endpoints such as the 6 Minute Walking Test (6MWT) have been shown to not be applicable in all paediatric age subsets, [3] and therefore may not be useful in elucidating patient capabilities.

COAs are a *well-defined and reliable assessment* of concepts of interest, which can be used in adequate, well-controlled studies in a specified context. COAs capture patient functionality and can be deployed through the use of wearable sensor technology; this feasibility study presents data obtained from patients with paediatric rare diseases who were assessed with this type of technology.

**Methods:** Niemann Pick-C (NP-C) (n = 10) and Duchenne Muscular Dystrophy (DMD) (n = 8) patients were asked to wear a wrist-worn wearable sensor at home for a minimum of 12 weeks. Feasibility was assessed qualitatively and quantitatively, with data captured in 30 minute epochs, measuring the mean of epoch’s with the most steps over a month (ADM), average daily steps (ADS), average steps per 30 minute epoch (ADE) (table 1) and reasons for non-adherence (table 2). No restriction in the minimum number of epochs available for analysis were applied, and all patient data analysed.

**Results:** Discrepancies in ambulatory capacity were observed between NP-C and DMD patients overall, with NP-C patients covering greater distances and taking more steps daily. Qualitative assessment of both patient groups highlighted their relationships with the technology, which in turn detailed adherence. Some patients exhibited behavioural issues which resulted in a loss of data and low engagement.

**Conclusions:** The wearable sensor technology was able to capture the ambulatory capacity for NP-C/DMD patients. Insights into disease specific parameters that differed were gained, which will be used for developing the technology further for use in future trials. Additional work is required to correlate the wearable device data with other clinical markers, however the study displays the feasibility of wearable sensors/apps as potential outcome measures in clinical trials.

**Table 1:** Summary demographic/ambulation data

	NP-C	DMD
**Demographics**	n = 10	n = 8
Age (median) in years	10	11
Age (range) in years	6–14	9–15
Sex M:F	6:4	8:0
**6MWT (m)**	n = 8	n = 8
Mean (SD)	444 (± 201.7)	322.4 (± 120.5)
Median	450	325
Range	109–772	178–522
**Mean values for wearable metrics at baseline**	n = 4	n = 6
Average daily maximum	1450.82	848.28
Average daily steps	9582.71	4504.66
Average daily steps per 30 min epoch	392.37	214

**Table 2:** Adherence issues summary

	Specific issue	Impact
**NP-C**	**Disease severity** Behavioural and advanced disease related (non-ambulation): Biting through strap **Mum of child with NP-C:** *“She loves the colour purple – it’s her favourite colour. But because of the soft texture of the strap she bites through it”.*	Low engagement Data loss Required frequent replacement
**DMD**	**Behavioural issues** Removing watch due to asymmetric design - touch on the skin **Specialist physio:** *“Some of our DMD boys didn’t like the asymmetry. It is quite large on the wrist and the strap may need a shorter alternative for young children”.*	Data loss

**Acknowledgements:** Duchenne UK, Niemann Pick Association UK & International Niemann Pick Disease Alliance (INPDA) for funding and support.

**Consent to publish:** Informed consent to publish has been obtained from patients.

**References**

1. Wang TJ, Tomasi PA, Bourgeois FT: Delays in completion and results reporting of clinical trials under the Paediatric Regulation in the European Union: A cohort study, *PLoS Med* 2018, 15: 10.1371/journal.pmed.1002520
2. Spotlight on rare diseases, *Lancet Diabetes Endocrinol* 2019, 7: 75
3. Ollivier C, Sun H, Amchin W, et al: New Strategies for the Conduct of Clinical Trials in Pediatric Pulmonary Arterial Hypertension: Outcome of a Multistakeholder Meeting With
4. Patients, Academia, Industry, and Regulators, Held at the European Medicines Agency on Monday, June 12, 2017. J Am heart Assoc 2019, 8: 10.1161/jaha.118.011306

### P16

#### Establishing a registry on rare congenital malformations in Germany

##### Andrea Schmedding^1^, Udo Rolle^1^, Jessica Vasseur^2^, Holger Storf^2^

###### ^1^Department of Pediatric Surgery and Pediatric Urology, University Hospital, Goethe University Frankfurt, Frankfurt am Main, Germany; ^2^Medical Informatics Group, University Hospital, Goethe University Frankfurt, Frankfurt am Main, Germany

**Correspondence:** Andrea Schmedding

*Orphanet Journal of Rare Diseases* 2020, **15(Suppl 1)**:P16

**Background:** Neonatal surgery is decentralized in Germany. In 2015 there were 89 departments of pediatric surgery that treated 93% of the abdominal wall defects with an average case load of less than 5 per unit [1]. Patient organizations stress the importance of quality measurements for the care of children with rare diseases.

**Study plan:** Currently, there is no nationwide data collection regarding the short term and long term care of patients with congenital malformations, who often need surgery during the first weeks of life. The German Society of Pediatric Surgery, which covers almost all of the 130 German pediatric surgical units, has initiated the work of creating a national patient registry (KiRaFe) for the following congenital malformations: Malformations of the gastrointestinal tract, the abdominal wall, the diaphragm, and meningomyelocele. The development of the registry involves three different patient organizations and health care professionals from all over Germany. The registry will be set up in 2020 based on the Open Source Registry System for Rare Diseases (OSSE).

The primary objective of the registry is the measurement of quality attributes of rare congenital malformations. Furthermore, the registry will facilitate recruitment of patients to clinical trials. It will also serve as a basis for policy making and planning of health and social services for people with rare disorders. Informed consent will be obtained from the participants. The registry will include core data, mainly comprising information on the set of malformations of each patient. Each malformation will then prompt further different modules for data collection. This modular structure offers the greatest possible flexibility for the documentation of patients with more than one congenital malformation. Data will be collected by health care professionals.

**Results:** Since the start of the preparation 47 individuals, either working in one of 27 hospitals or being member of one of the three patient organizations, have contributed in the ongoing activities. The registry is listed in the European Directory of Registries (ERDRI) [2]. Ethical approval was obtained, financial resources were secured. In 2020, 83 German hospitals and three non-German hospitals confirmed their intention to document their patients within the registry.

**Conclusion:** The registry is an example for a nationwide collaboration with the goal to optimize the quality of care for a patient group with rare diseases.

**Acknowledgements:** We thank the Dr. Emil Alexander Huebner und Gemahlin-Stiftung and the German Society of Pediatric Surgery for the financial support of the registry.

We thank the KiRaFe-Group for the work on the registry: Bahr, Micha; Clemen, Christian; Eismann, Daniel; Fuchs, Jörg; Gitter, Heidrun; Gradhand, Elise; Grasshoff-Derr, Sabine; Großer, Kay; Günther, Patrik; Hemminghaus, Michael; Hubertus, Jochen; Jechalke, Stephan; Jenetzki, Ekkehart; Kirschner, Hans Joachim; Klein, Tobias; König, Tatjana; Krause, Monika; Ludwikowski, Barbara; Luithle, Tobias; Märzhäuser, Stefanie; Michel, Armin-Johannes; Moursi, Ahmed Gamal Abdelmalek; Müller, Annette; Pfleiderer, Oliver; Rohleder, Stephan; Rolle, Udo; Rothe, Karin; Schäfer, Mattias; Schaible, Thomas; Schmedding, Andrea; Schmiedeke, Eberhard; Schmittenbecher, Peter; Schnekenburger, Franz Georg; Schulze, Annekatrin; Schuster, Tobias; Schwarzer, Nicole; Siebert, Julia; Storf, Holger; Tomuschat, Christian; Vasseur, Jessica; Vierling, Christian; Weltzien, Alexandra; Wessel, Lucas; Widenmann-Grolig, Anke; Wirmer, Hanno; Zerche, Arnim; Ziegler, Anna-Maria

**References**

1. Schmedding A, Rolle U. Decentralized Rather than Centralized Pediatric Surgery Care in Germany. Eur J Pediatr Surg 2017; 27: 399–406
2. ERDRI.dor - European Directory of Registries [https://eu-rd-platform.jrc.ec.europa.eu/erdridor/register/4505], accessed 30th june, 2020

### P17

#### Improving communication about clinical trials in Cystic Fibrosis: a starting point for plain language summaries

##### Hilde De Keyser^1^, Emmanuelle E Bardin^1, 2^, Jill Bonjean^1^, Fiona Dunlevy^3^, Rebecca Brendell^4^, Lorna Allen^4^

###### ^1^Cystic Fibrosis Europe, Brussels, Belgium; ^2^Association Muco, Brussels, Belgium; ^3^Quality department, European Cystic Fibrosis Society-Clinical Trials Network, Leuven, Belgium; ^4^UK Cystic Fibrosis Trust, London, UK

**Correspondence:** Hilde De Keyser - hilde.dekeyser@cf-europe.eu

*Orphanet Journal of Rare Diseases* 2020, **15(Suppl 1)**:P17

Cystic Fibrosis Europe (CF Europe) is the federation of national CF associations in Europe. To represent and defend the interests of people with CF, we engage with key stakeholders such as the European Union and industry. The Cystic Fibrosis Round Table of Companies (CFRToC) is a collaboration between CF Europe and five pharmaceutical companies (to date). Through biannual meetings, we aim to institute a long-term educational collaboration with companies with an interest in CF. Membership of industrial partners is dependent upon adherence to the CFRToC Code of Conduct and a financial contribution for CF Europe to fulfil its missions.

Common objectives include access to information. One strong example, applicable even beyond rare diseases, is the need for improved communication regarding clinical trials (CTs) which has been inconsistent and often difficult to understand. From 2021, the new European CT regulation 536/2014 will oblige sponsors to share CT results through lay summaries. To help move this initiative forward, CF Europe, with the active support of the Cystic Fibrosis Trust, is collaborating with the European Cystic Fibrosis Society-Clinical Trials Network (ECFS-CTN) and CFRToC members to establish a glossary of relevant CF terms. It will be freely available so that all stakeholders can systematically use it in patient-friendly scientific summaries and wider communication.

In a pilot project, people with CF and patient associations, together with industrial partners will shortlist 10 terms. These will be defined by lay members and subsequently subjected to the study and approval of the legal department of participating companies. Provided this process is successful, we aim to create 30 approved definitions by the end of 2020. CF Europe and ECFS-CTN intend to advertise the use of this glossary online and through communications at scientific events. National patient organisations will be further encouraged to provide translations in their national language.

### P18

#### Setting up an infra-structure of healthcare for Alkaptonuria in Germany by dissemination and networking

##### Leona Wagner^1^, Harald Wilke^1^, Marion Hoyer^1^, Gerhard Meng^2^, Konstantinos Kolokotronis^2^, Stephan vom Dahl^3^, Anibh Das^4^, Athanasia Ziagaki^5^

###### ^1^Deutschsprachige Selbsthilfegruppe für Alkaptonurie e.V., Stuttgart, Germany; ^2^Institute of Human Genetics, Biocenter, Julius-Maximilians-University Würzburg, Würzburg, Germany; ^3^Department of Gastroenterology, Hepatology and Infectious Diseases, Heinrich-Heine University, Düsseldorf, Germany; ^4^Clinic for Pediatric Kidney, Liver, and Metabolic Diseases, Hannover Medical School, Hannover, Germany; ^5^Interdisciplinary Centre of Metabolism: Endocrinology, Diabetes and Metabolism, Charité-University Medicine Berlin, Berlin, Germany

*Orphanet Journal of Rare Diseases* 2020, **15(Suppl 1)**:P18

Alkaptonuria (AKU, ochronosis) is an inborn metabolic disease, resulting in the accumulation of the metabolic intermediate homogentisic acid (HGA). Oxidation of HGA by air or within connective tissue causes darkening of the urine, pigmentation of eyes and ears, kidney- and prostrate-stones, aortic stenosis, but most severely an early onset of arthritis called ochronotic arthropathy (ochronosis) due to deposition in the cartilage. Ochronosis is very painful, disabling and progresses rapidly. Starting in the thirties with the spine and affecting large joints in the forties, patients frequently require joint replacements in their fifties and sixties [1,2]. Like many of the rare diseases, AKU-patients undergo a long odyssey of several years until their diagnosis.

The German AKU-Society “Deutschsprachige Selbsthilfegruppe für Alkaptonurie (DSAKU) e.V.” was founded in 2012 and became subsequently registered as a non-profit patient organization. First of all, the DSAKU identified AKU-patients, set up a homepage **[3]** and designed flyers with information for patients, their families, medical professionals and healthcare services. Second, it offered workshops on AKU-related issues and enabled personal exchange. Third, it raised awareness of AKU, both nationally and internationally by information booths, presentations and posters at scientific congresses as well as rare disease days (RDD). Fourth, in response to the needs of patients, it established collaborations and built up national networks for a better health care accordingly. Thus, patients were encouraged to visit the centers for metabolic diseases at the Charité (Berlin), Hannover Medical School (MHH), University of Düsseldorf and Institute of Human Genetics at the University of Würzburg to bundle knowledge and expertise. The DSAKU is member of ACHSE e.V., NAKOS, EURORDIS and MetabERN and registered in the databases SE-Atlas, ZIPSE and Orphanet. Finally, the DSAKU is nationally and internationally active in health politics regarding training in drug safety and evidence-based medicine.

**References**

1. Phornphutkul C, Introne W, Perry M, Bernardini I, Murphey M, Fitzpatrick D, Anderson, P D, Huizing M, Anikster Y, Gerber L H, Gahl W A: Natural history of alkaptonuria. *N Engl J Med* 2002, 347:2111–2121.
2. Ranganath L, Jarvis, J, Gallagher J: Recent advances in management of alkaptonuria (invited review; best practice article) *J Clin Pathol* 2013, 66:367–373.
3. Startseite [Internet]. Dsaku.de. 2020 [accessed 28 June]. Available at: http://www.dsaku.de/

### P19

#### Development of an app for the management of autoinflammatory diseases using an innovative patients-clinicians codesign approach

##### Silvia Federici^1^, Camilla Santu^2^, Marta De Munari^2^, Antonella Insalaco^1^, Camilla Celani^1^, Luca De Roberto^2^, Giuseppe Ciniero^2^, Fabrizio De Benedetti^1^, Ruggero Di Maulo^2^

###### ^1^Rheumatology, Bambino Gesù Hospital, Rome, Italy; ^2^Cloud-R s.r.l., Milan, Italy

**Correspondence:** Silvia Federici - silvia.federici@opbg.net; Camilla Santu - camilla.santu@cloud-r.eu

*Orphanet Journal of Rare Diseases* 2020, **15(Suppl 1)**:P19

**Introduction:** Autoinflammatory diseases are rare conditions characterized by recurrent episodes of inflammation with fever associated to elevation of acute phase reactants and symptoms affecting mainly the mucocutaneous, musculoskeletal or gastrointestinal system. These diseases affect the quality of life of patients and their families.

**Objectives:** Aim of this project is to develop a tool able to ameliorate patients’ management of the disease and to enhance patient-physician communication.

**Methods:** To develop a tool based on real-life needs, we involved patients and caregivers since the initial phase of the project. A first workshop designed to capture their needs was organized. Innovative co-design activities were performed through “LegoSeriousPlay™” (LSP) methodology[1–3].

During a first phase of “divergence” 13 patients (from teen-agers to adults) affected by different AIDs (FMF, TRAP, CAPS, MKD) and 2 physicians where involved in the LSP activities. Participants were asked to describe, through LEGO and metaphors:

- The disease
- Themselves in comparison with the disease
- Solutions and supports which could help them in managing the disease

After each step the participants presented their models, and everyone was engaged in the discussion. The ideas collected during the three phases allowed to make a list of functionalities identified as necessary for the app to be developed.

Due to the actual Sars-CoV-2 sanitary emergency the second phase of the project, aimed at presenting the participants the results of the first meeting and proceed with the App finalization was performed through web- based meeting and surveys in which the patients and caregivers actively participated.

**Results:** In the first phase patients and caregivers participated actively expressing various needs, that we subsequently summarized in 4 main areas (Table 1). Participants were then further involved and their opinion taken into consideration for the User Experience and Interface definition for the development of the Mobile App including the required functionalities (after a further activity of prioritization).

**Table 1:** Needs expressed by participants

Area	Request	N°
Patient’s information-diary	Fever attacks, symptoms registration Repository of health information	12 5
Community	Online communities - patient to patient Direct connection with physicians	14 11
Personal agenda	Calendar: therapy, visits, exam scheduling alerts (appointments, deadlines, reminders)	13 4
Clinical/practical information	Disease information Legal information-patients’ rights	20 7

**Conclusions:** Our project shows that active involvement of patients and caregivers in the design of a mobile-App can be achieved through innovative approaches. The objective is to obtain an App tailor-made on the real patients’ needs and a consequent high satisfaction and long-term adoption of the tool.

**Acknowledgments:** Project promoted and funded by Novartis. Special thanks to the patients and parents of patients who participated in the project with enthusiasm, contributing to the definition of the App.

**Disclosure of interest:** None declared.

**References**

1. Rasmussen, R., When you build in the world, you build in your mind. Design Management Review, 2006, 56–63.
2. Frick, E. Tardini, S. Cantoni L., White Paper on LEGO SERIOUS PLAY. Università della Svizzera Italiana, 2013
3. Kristiansen, P.Hansen, P., Nielsen, L. Articulation of tacit and complex knowledge. In P. Schönsleben, M. Vodicka, R. Smeds, & J. Ove Riis (Eds.). 13th International Workshop of the IFIP WG 5.7 SIG, (p. 77–86)

### P20

#### Project HERCULES: Overcoming the HTA hurdle through patient led collaboration

##### E. Crossley^1^, F. Chandler^2^, J. Godfrey^3^, K. Abrams^4^, J. Mumby-Croft^5^, J. Carlton^6^, P. A. Powell^6^, J. O’Hara^7^

###### ^1^Duchenne UK, London, UK; ^2^Sanofi-Genzyme, London, UK; ^3^JG Zebra Consulting, London, UK; ^4^University of Leicester, Leics, UK; ^5^Source Health Economics, Oxford, OXF, UK; ^6^SChARR, University of Sheffield, Sheffield, UK; ^7^HCD Economics, Daresbury, UK

**Correspondence:** E. Crossley

*Orphanet Journal of Rare Diseases* 2020, **15(Suppl 1)**:P20

Access to new treatments for Duchenne muscular dystrophy (DMD) is a challenging hurdle, with little useful evidence fully reflecting patient experience. The UK patient group Duchenne UK recognised that limited evidence and tools for demonstrating the value of treating DMD were available to support access decisions for these new treatments, and adopting an innovative collaborative approach, launched Project HERCULES. This international multi-stakeholder collaboration has developed a core suite of patient focused disease level tools suitable for Health Technology Assessment; including a natural history model, a bespoke Quality of Life measure, a burden of illness study and a disease level economic model. The consortium includes numerous global patient organisations, nine pharmaceutical companies, world-leading health economists, academics, clinicians, Health Technology Assessment bodies and other expert advisers.

Patient communities are not simply the subject of research, they can in fact direct research, ensuring the lived experience of a condition informs every aspect of health economics and outcomes research leading to results that better reflect the true impact of that condition and demonstrating value in treatments that can delay or halt progression. The leadership of a patient organisation ensures that Project HERCULES’ focus is on the patient, enables access to data sources and expertise which may be inaccessible for individual researchers and has enabled a bottom up approach to HTA evidence.

This has led to the identification of a newly defined health state for DMD, a stage in which patients are unable to walk but still able to weight bear. This health state was hugely important to the quality of life of patients and families as well as to the burden of illness on patients, families and health and social care.

This approach also ensured the creation of a measure for Quality of Life in DMD that identified issues important to patients that were not well recognised by clinicians and a burden of illness study that identified those elements of DMD that have the biggest impact on patients and families.

The Project HERCULES approach to patient engagement could provide a model for other rare disease communities looking to prepare for new treatments in their disease area.

### P21

#### Improving patient communication and education using POC (Point of Care) channel

##### Marta Fonfría^1^, Joima Panisello^2^, Amelia Carro^2^, Xavier Lleixà^2^

###### ^1^CREER. IMSERSO, Burgos, Spain, 09001; ^2^DigimEvo, Barcelona, Spain, 08014

**Correspondence:** Xavier Lleixà

*Orphanet Journal of Rare Diseases* 2020, **15(Suppl 1)**:P21

**Introduction:** Gaps in communication and education are becoming one of the biggest key pain points for patients that are suffering rare diseases. Due to the limited resources and the misleading information on the internet we wanted to test the POC systems to deliver more efficiently the information to our patients and their relatives. User-friendly information at the point of care should be well structured, rapidly accessible, and comprehensive.

**Method:** We implemented a specific POC channel using several touchpoints to deliver the right content at the right time. We created and selected the video content that will be most helpful to our patients. Later on, we analyzed the patient journey and we decided to use a mobile app where the patients could search for information when they are at their home. At the medical practice, we use the waiting room and exam room as learning areas through monitors and tablets. Moreover, healthcare professionals are prescribing content to their patients that they reviewed when they are home.

**Results:** Thanks to the use of the POC channel and technologies related we were able to reduce the time needed to perform an explanation by 35%. Furthermore, our healthcare professionals reported that their conversations with the patients improved 50% and patient satisfaction increased by 60%.

**Conclusion:** POC channel created a positive impact on our patient experience allowing us to be more efficient delivering the information to our patients and their relatives.

### P22

#### Blood-Brain Barrier Permeabilization with Engineered Tumor Necrosis Factor-α Followed by R-CHOP is an Active and Safe Salvage Therapy in Primary CNS Lymphoma

##### Teresa Calimeri^1^, Maurilio Ponzoni^2,3^, Flavio Curnis^4^, Gian Marco Conte^5^, Eloise Scarano^6^, Eltjona Rrapaj^4^, Daniela De Lorenzo^6^, Dario Cattaneo^7^, Federico Fallanca^8^, Alessandro Nonis^2^, Marco Foppoli^1^, Paolo Lopedote^2^, Giovanni Citterio^1^, Letterio S. Politi^5^, Marianna Sassone^1^, Piera Angelillo^1^, Elena Guggiari^1^, Sara Steffanoni^1^, Vittoria Tarantino^1,9^, Fabio Ciceri^2,10^, Claudio Bordignon^11^, Nicoletta Anzalone^2,5^, Angelo Corti^2,4^, Andrés J. M. Ferreri^1^

###### ^1^Lymphoma Unit, Department of Onco-Hematology, IRCCS San Raffaele Scientific Institute, Milano, Italy; ^2^Università Vita-Salute San Raffaele, Milano, Italy; ^3^Pathology Unit; IRCCS San Raffaele Scientific Institute, Milano, Italy; ^4^Division of Experimental Oncology, Tumor Biology and Vascular Targeting Unit, IRCCS San Raffaele Scientific Institute, Milano, Italy; ^5^Neuroradiology Unit, IRCCS San Raffaele Scientific Institute, Milano, Italy; ^6^Datamanager and Study Coordinator Office, Lymphoma Unit, IRCCS San Raffaele Scientific Institute, Milano, Italy; ^7^Unit of Clinical Pharmacology, Department of Laboratory Medicine, ASST Fatebenefratelli Sacco University Hospital, Milano, Italy; ^8^Nuclear Medicine Unit, IRCCS San Raffaele Scientific Institute, Milano, Italy; ^9^PhD Program in Clinical and Experimental Medicine, University of Modena and Reggio Emilia, Modena, Italy; ^10^Hematology and BMT Unit, Department of Onco-Hematology, IRCCS San Raffaele Scientific Institute, Milano, Italy; ^11^MolMed SpA, Milano, Italy

**Correspondence:** Andrés J. M. Ferreri - ferreri.andres@hsr.it

*Orphanet Journal of Rare Diseases* 2020, **15(Suppl 1)**:P22

**Background:** R-CHOP is the standard treatment of diffuse large B-cell lymphoma (DLBCL). Primary DLBCL of the CNS (PCNSL) is an exception because of the incapability of related drugs to cross the blood-brain barrier. TNF-α fused to the NGR peptide targets CD13^+^ vessels and enhances vascular permeability, providing the rationale for using R-CHOP in PCNSL patients.

**Patients and methods**: In this phase II trial, we addressed activity and safety of R-CHOP preceded by low-dose NGR-hTNF in patients with relapsed/refractory PCNSL. Overall response rate was the primary endpoint. NGR-hTNF/R-CHOP would be declared active if ≥ 12 responses were recorded among 28 assessable patients. Secondary endpoints included safety, predictive value of NGR-hTNF inhibitors (chromogranin A; soluble TNF receptors), and expression of CD13 by PCNSL vessels.

**Results:** 28 heavily pretreated patients (median age 58 years, range 26–78; 14 males) were enrolled and written informed consent was obtained from each of them. Low-dose NGR-hTNF exerted relevant effects on vascular permeability specifically in tumor and peritumoral areas as shown by standardized DCE-MRI, SPECT, and plasma/CSF pharmacokinetics studies. NGR-hTNF/R-CHOP combination was active, with confirmed tumor response in 21 patients (75%; 95% CI = 59–91%), which was complete in 11. At a median follow-up of 18 (12–29) months, five patients remain relapse-free and six are alive. Treatment was well tolerated, without dose reductions or interruptions. High plasma levels of chromogranin A were associated with proton pump inhibitors use and lower remission rate. CD13 was expressed by both pericytes and endothelial cells of PCNSL.

**Conclusions:** NGR-hTNF/R-CHOP is active and safe in patients with relapsed/refractory PCNSL. Its activity is in line with the broad expression of CD13 in tumor vessels. Proton pump inhibitors should be avoided during TNF-based therapy. This innovative approach deserves to be addressed as first-line treatment in PCNSL patients.

Funded by The Leukemia and Lymphoma Society; INGRID trial; EudraCT number 2014-001532-11; ClinicalTrials.gov number, NCT03536039.

**Acknowledgments:** The authors appreciate the excellent technical assistance and sustained scientific collaboration of Angelo Diffidenti and Maria Colia (Research Nurses of the Lymphoma Unit, San Raffaele Scientific Institute, Milan, Italy), Stefano Orezzi (Neuroradiology Unit, San Raffaele Scientific Institute), Daniela De Lorenzo (Datamanger and Study Coordinator Office of the Lymphoma Unit, San Raffaele Scientific Institute), Anna Chiara (Radiotherapy and Tomotherapy Unit, San Raffaele Scientific Institute), and Elisabetta Miserocchi and Giulio Modorati (Ophthalmology Unit, San Raffaele Scientific Institute). They also acknowledge the hematologists and oncologists of the Department of Onco-Hematology, San Raffaele Scientific Institute, for their excellent clinical assistance. The authors are indebted to the enrolled patients and their families of the INGRID trial for their generous commitment.

The INGRID trial was performed without commercial funding. It was supported by a grant from the Leukemia and Lymphoma Society (A.J.M.F., grant 6510-17) and, in part, by a grant from the Associazione Italiana Ricerca control il Cancro (A.C., grant IG-19220). NGR-hTNF was kindly provided by Molmed SpA (Milan, Italy).

### P23

#### Preliminary real-world treatment patterns and outcomes in patients with spinal muscular atrophy (SMA) collected from the RESTORE registry

##### Laurent Servais^1^, John W. Day^2^, Darryl C. De Vivo^3^, Janbernd Kirschner^4^, Eugenio Mercuri^5^, Francesco Muntoni^6^, Perry B. Shieh^7^, Eduardo Tizzano^8^, Isabelle Desguerre^9^, Susana Quijano-Roy^10^, Kayoko Saito^11^, Marcus Droege^12^, Omar Dabbous^12^, Ankita Shah^12^, Melissa Menier^12^, Deepa Chand^12^, Frederick A. Anderson^13^

###### ^1^MDUK Oxford Neuromuscular Centre, University of Oxford, Oxford, United Kingdom; ^2^Department of Neurology, Stanford University Medical Center, Stanford, CA, United States; ^3^Departments of Neurology and Pediatrics, Columbia University Irving Medical Center, New York, NY, United States; ^4^Department of Neuropediatrics, University Hospital Bonn, Bonn, Germany; ^5^Department of Paediatric Neurology and Nemo Clinical Centre, Catholic University, Rome, Italy; ^6^Department of Developmental Neuroscience, University College London, London, United Kingdom; ^7^Department of Neurology, David Geffen School of Medicine at UCLA, Los Angeles, CA, United States; ^8^Department of Clinical and Molecular Genetics, Hospital Valle Hebron, Barcelona, Spain; ^9^Hôpital Necker Enfants Malades, APHP, Paris, France; ^10^Garches Neuromuscular Reference Center (GNMH), APHP Raymond Poincare University Hospital (UVSQ Paris Saclay), Garches, France; ^11^Institute of Medical Genetics, Tokyo Women’s Medical University, Tokyo, Japan; ^12^AveXis, Inc., Bannockburn, IL, United States; ^13^Center for Outcomes Research, University of Massachusetts Medical School, Worcester, MA, United States

**Correspondence:** Marcus Droege

*Orphanet Journal of Rare Diseases* 2020, **15(Suppl 1)**:P23

**Background:** Until recently, supportive care was the sole treatment option for SMA patients. Although 2 therapies are currently FDA approved (onasemnogene abeparvovec and nusinersen) [1,2], real-world safety and efficacy data are limited – particularly for patients who receive > 1 treatment. We report initial data from the RESTORE Registry, including cohort clinical characteristics, treatments received, and outcomes.

**Materials and methods:** RESTORE is a prospective, multicenter, treatment-agnostic registry of SMA patients. The primary objectives include assessment of contemporary SMA treatments; secondary objectives include assessment of healthcare resource utilization, caregiver burden, and changes in patient functional independence over time. Planned follow-up is 15 years from enrollment.

**Results:** As of 31 January 2020, data were available for 67 patients, all from *de novo* clinical sites in the United States; information on treatment regimens was available for 56 patients (Table 1).

**Table 1:** Demographics and clinical characteristics

Characteristic	Treatment-evaluable patients (N = 56) n (%)
*Gender*	49 (87.5)^a^
Female	29 (59.2)
Male	20 (40.8)
*SMN2 copy number*	53 (94.6)^a^
1	2 (3.8)
2	30 (56.5)
3	15 (28.3)
4	6 (11.3)
>4	0
*SMA type*	56 (100)^a^
Presymptomatic	5 (8.9)
I	38 (67.8)
II	8 (14.3)
III	5 (8.9)
*Age at first treatment*	43 (76.8)^a^
0–6 months	26 (60.5)
> 6–24 months	12 (27.9)
> 24 months	5 (11.6)

^a^Percentage based on eligible patients, n/N (%)

Disease-modifying treatments were administered sequentially or in combination. 91% of treated patients showed symptoms at SMA diagnosis, with the most common being hypotonia and limb weakness (Table 2).

**Table 2:** SMA symptoms at diagnosis of treated patients

	Treatment received					
Symptom, n (%)	Onasemnogene abeparvovec → nusinersen (n = 2)	Nusinersen → onasemnogene abeparvovec (n = 17)	Nusinersen → onasemnogene abeparvovec → nusinersen (n = 8)	Onasemnogene abeparvovec only (n = 18)	Nusinersen only (n = 11)	All treated patients (N = 56)
Patients with symptoms, n	2	16	8	15	10	51
Hypotonia	1 (50.0)	12 (75.0)	8 (100.0)	13 (86.7)	7 (70.0)	41 (80.4)
Limb weakness	2 (100.0)	13 (81.3)	7 (87.5)	11 (73.3)	9 (90.0)	42 (82.4)
Pneumonia or respiratory symptoms	2 (100.0)	10 (62.5)	5 (62.5)	5 (33.3)	1 (10.0)	23 (45.1)
Tongue fasciculations	0	10 (62.5)	3 (37.5)	6 (40.0)	0	19 (37.3)
Developmental delay	1 (50.0)	8 (50.0)	5 (62.5)	8 (53.3)	5 (50.0)	27 (52.9)
Constipation	1 (50.0)	5 (31.3)	4 (50.0)	2 (13.3)	4 (40.0)	16 (31.4)
Swallowing or feeding difficulties	1 (50.0)	12 (75.0)	7 (87.5)	8 (53.3)	1 (10.0)	29 (56.9)
Other	0	0	0	0	2 (20.0)	2 (3.9)

Ten patients had > 1 CHOP INTEND score available for analysis and 8 (80%) had increased scores during the initial follow-up period. Data for CHOP INTEND changes by treatment regimen were not yet available. Adverse event (AE) data were reported for 39 of the 56 patients with known treatment regimens (Table 3); 32 (82.1%) reported ≥ 1 AE; 15 (38.5%) reported ≥ 1 serious AE (6 [15.4%] related to treatment).

**Table 3:** Treatment-emergent AEs by treatment

	Treatment received					
	Patients receiving > 1 active therapy			Patients receiving monotherapy		
Adverse event, n (%)	Onasemnogene abeparvovec → nusinersen (n = 1)	Nusinersen → onasemnogene abeparvovec (n = 14)	Nusinersen → onasemnogene abeparvovec → nusinersen (n = 8)	Onasemnogene abeparvovec only (n = 12)	Nusinersen only (n = 4)	Total (n = 39)
≥1 treatment-emergent AE (any grade)	1 (100.0)	11 (78.6)	8 (100.0)	10 (83.3)	2 (50.0)	32 (82.1)
Serious AEs	1 (100.0)	4 (28.6)	5 (62.5)	4 (33.3)	1 (25.0)	15 (38.5)
Serious and related AEs^a^	0	3 (21.4)	2 (25.0)	1 (8.3)	0	6 (15.4)
AEs of special interest to onasemnogene abeparvovec						
Hepatotoxicity^b^	0	8 (57.1)	6 (75.0)	6 (50.0)	0	20 (51.3)
Thrombocytopenia^c^	0	3 (21.4)	0	1 (8.3)	0	4 (10.3)
Cardiac AEs^d^	1 (100.0)	3 (21.4)	3 (37.5)	2 (16.7)	0	9 (23.1)

^a^For patients who received > 1 therapy, relatedness could be due to either medicinal product and was not specified by the reporter. ^b^Primarily transient transaminase elevations. ^c^Primarily isolated decrease in platelet count without clinical evidence of bleeding events. ^d^Cardiac AEs include bradycardia, troponin elevations, cardiac arrest, thrombotic microangiopathy, and hepatomegaly; at least 1 blood and lymphatic system-related AE and 1 event of hepatomegaly were misclassified as cardiac and will be corrected

The RESTORE Registry continues to enroll new patients and activate new sites.

**Conclusion:** The current enrollment in the RESTORE Registry reflects a range of SMA patient types and treatment regimens. Limitations of this analysis include a small number of patients; short, variable duration of follow-up; and variable completeness of data across study sites at time of analysis. Since only AEs that occur after enrollment are recorded, AEs occurring soon after initiation of treatment may not be captured. Most patients with > 1 CHOP INTEND evaluation achieved higher scores over the initial follow-up period. Based on limited data available, AE experience of onasemnogene abeparvovec observed in the RESTORE Registry is consistent with experience in clinical trials for SMA; no new safety signals were identified among patients treated with onasemnogene abeparvovec or among those who switched treatments.

**References**

1. Spinraza (nusinersen) [package insert]. Cambridge, MA; Biogen, Inc.; June 2019.
2. Zolgensma (onasemnogene abeparvovec-xioi) [package insert]. Bannockburn, IL; AveXis, Inc.; May 2019.

### P24

#### Educational consequences of rare conditions – development of an observation instrument

##### Gunilla Jaeger^1^, AnnCatrin Röjvik^1^, Kerstin W. Falkman^2^, Erland Hjelmquist^2^

###### ^1^Ågrenska, Gothenburg, Sweden; ^2^Department of Psychology, University of Gothenburg, Gothenburg, Sweden

**Correspondence:** Gunilla Jaeger - gunilla.jaeger@agrenska.se

*Orphanet Journal of Rare Diseases* 2020, **15(Suppl 1)**:P24

Ågrenska, a Swedish national centre for rare diseases, has for thirty years arranged courses for families of children with rare diagnoses and has experienced that the conditions often have complex and varying consequences in the children ´s everyday lives. Knowledge of these consequences and of how to adapt the treatment, environment and activities to create the best possible conditions for participation and learning, is often lacking. Many professionals also report lack of sources of knowledge. Knowledge formation and dissemination are thus of outmost importance.

In order to aid knowledge formation and dissemination Ågrenska has developed an observation instrument for children with rare diagnoses, identifying both abilities and difficulties on a group level. The instrument consists of 144 quantitative and 71 qualitative items and covers ten areas: social/communicative ability, emotions and behaviours, communication and language, ability to manage his/her disability and everyday life, activities of daily life, gross and fine motor skills, perception and worldview, prerequisites for learning and basic school abilities.

Observations are made during the children ´s school and pre-school activities during the Ågrenska course. Teachers and special educators, working with the children, are responsible observers.

Some school-related abilities are difficult to observe during the five-day stay. This information is instead collected through a telephone interview with the children ´s home teacher.

The instrument was content validated against a number of existing instruments. The items were considered relevant as they, with few exceptions, appear in well-known assessment tools.

To test interrater reliability observations of six children were performed. Each child was observed by two educators. Interrater reliability was calculated for the 116 quantitative items usually observed during the course. Interrater reliability reached 92.5%.

### P25

#### Cost-effectiveness analysis of newborn screening for spinal muscular atrophy (SMA) in the United States

##### Ramesh Arjunji^1^, Jenny Zhou^2^, Anish Patel^1^, Marie Louise Edwards^3^, Michael Harvey^2^, Eric Wu^4^, Omar Dabbous^1^

###### ^1^AveXis, Inc., Bannockburn, IL, United States; ^2^Analysis Group, London, United Kingdom; ^3^Analysis Group, New York, NY, United States; ^4^Analysis Group, Boston, MA, United States

**Correspondence:** Anish Patel

*Orphanet Journal of Rare Diseases* 2020, **15(Suppl 1)**:P25

**Background:** SMA is a neurodegenerative disease caused by survival motor neuron 1 gene (*SMN1*) deletion or mutation [1, 2]. Disease severity (SMA type) correlates with *SMN2* copy number [1, 2]. Gene therapy with onasemnogene abeparvovec provides sustained, continuous production of SMN protein, and is FDA approved [3], with ongoing trials for SMA type 2 (SMA2) and SMA3, and pre-symptomatic treatment for all SMA types. With treatment options available, many states in the United States (US) are implementing newborn screening (NBS) to detect *SMN1* deletions and *SMN2* copies, providing early diagnosis and the option of pre-symptomatic treatment [4]. We examine the economic consequences of implementing NBS for SMA and pre-symptomatic treatment with onasemnogene abeparvovec gene therapy among newborns in the US.

**Materials and methods:** A decision-analytic model was built to assess the cost effectiveness of NBS in 10,000 hypothetical newborns from a US third-party payer perspective. The model included 3 separate arms, each allowing for a different treatment strategy. Model inputs for epidemiology, test characteristics, and screening and treatment costs were based on publicly available literature (Table 1). Inputs and assumptions of lifetime costs and utilities for SMA types were obtained from the 2019 Institute for Clinical and Economic Review SMA report [5]; other values were sourced from published literature. Model outputs included total costs, quality-adjusted life years (QALYs), and incremental cost-effectiveness ratios (ICERs). Scenario and sensitivity analyses tested model robustness.

**Table 1:** Model Inputs

Input^a^	Value	Source
SMA birth prevalence	9.4/100,000	Lally et al., 2017 [6]
*SMN1* deletion/*SMN1* point mutation	95.00%/5.00%	Kraszewski et al., 2018 [7]; Chien et al., 2017 [8]
*SMN2 copies and conditional SMA type distribution*		
*SMN2* – 2 copies (SMA Type I/II/III)	45.00% 78.88%/16.48%/4.64%	Vill et al., 2019 [9]; Calucho et al., 2018 [10]
*SMN2* – 3 copies (SMA Type I/II/III)	19.00% (14.74%/54.27%/30.99%)	Vill et al., 2019 [9]; Calucho et al., 2018 [10]
SMN2 - 4 copies (SMA Type I/II/III)	36.00% (0.58%/11.41%/88.01%)	Vill et al., 2019 [9]; Calucho et al., 2018 [10]
*SMA type distribution - undetected SMA or SMN1 point mutation*		
Type I/II/III	58.00%/29.00%/13.00%	Lally et al., 2017 [6]
SMA NBS cost per newborn	$10	Assumption
Reflex screening (per newborn with SMA-positive results from initial screening)	$20	Assumption
Onasemnogene abeparvovec drug cost	$2,125,000	Red book 2019 [11]
Onasemnogene abeparvovec symptomatic administration^b^	$141	CMS physician fee schedule 2018 [12]; Red book 2018 [13]
Onasemnogene abeparvovec pre-symptomatic administration^b^	$125	CMS physician fee schedule 2018 [12]; Red book 2018 [13]

*AAV9* adeno-associated virus serotype 9, *CMS* Centers for Medicare & Medicaid Services, *NBS* newborn screening, *SMA* Spinal Muscular Atrophy, *SMN* survival motor neuron, *USD* United States dollar

^a^Costs are reported in 2019 USD. ^b^Administration costs include intravenous infusion, anti-AAV9 diagnosis test (symptomatic treatment only), laboratory monitoring and prednisolone

**Results:** In the base case of NBS for 10,000 newborns, no SMA screening and symptomatic treatment for SMA1 generated 269,988 QALYs for a total treatment cost of $2,628,116 over a lifetime horizon. SMA NBS and pre-symptomatic treatment with onasemnogene abeparvovec for positive tests incurred a total cost of $3,150,087 and produced 269,997 QALYs. The majority of the costs were attributable to treatment. The incremental cost per QALY gained was $57,969/QALY when compared to no SMA screening. If only pre-symptomatic patients with ≤ 3 *SMN2* copies were treated, the SMA NBS strategy cost $2,485,813 and produced 269,996 QALYs. This was dominant when compared to no SMA screening, producing more QALYs at a lower cost (Table 2).

**Table 2:** Base-case results (per 10,000 newborns)

	Arm 1: No SMA screening + Symptomatic treatment for SMA	Arm 2: SMA NBS + Pre-symptomatic treatment for SMA	Arm 3: SMA NBS + Pre-symptomatic treatment for SMA with ≤ 3 SMN2 gene copies
*Costs (2019 USD)*			
Screening costs	NA	$100,000	$100,000
Reflex test costs	NA	$31	$31
Treatment costs	$2,628,116	$3,050,056	$2,385,782
Total costs	$2,628,116	$3,150,087	$2,485,813
*Effectiveness*			
QALYs	269,987.67	269,996.67	269,996.09
**Incremental results**		**Arm 2 vs. Arm 1**	**Arm 3 vs. Arm 1**
Incremental costs	–	$521,971	-$142,303
Incremental QALYs	–	9.00	8.42
Incremental costs per QALY gained	–	$57,969	Dominant

*NA* not applicable, *NBS* newborn screening, *QALYs* Quality-Adjusted Life Years, *SMA* spinal muscular atrophy, *SMN* survival motor neuron, *USD* United States Dollar

**Conclusion:** At a willingness-to-pay threshold of $150,000 per QALY, adoption of NBS with SMA screening and allowing patients to receive effective pre-symptomatic gene therapy represents a cost-effective option for US payers. The results were most sensitive to treatment strategies (i.e. treatment depending on *SMN2* copy number) and distribution of SMA types; screening costs had minimal impact.

**References**

1. Mercuri E, Bertini E, Iannaccone ST: Childhood spinal muscular atrophy: controversies and challenges, *Lancet Neurol*, 2012, 5: 443–52.
2. Kolb SJ, Kissel JT: Spinal muscular atrophy, *Neurol Clin*, 2015, 4: 831–846.
3. Zolgensma (onasemnogene abeparvovec-xioi) [package insert]. Bannockburn, IL; AveXis, Inc.; May 2019.
4. Recommended Uniform Screening Panel, 2018. Available from: https://www.hrsa.gov/advisory-committees/heritable-disorders/rusp/index.html. (Accessed April 3, 2020).
5. Institute for Clinical and Economic Review, 2019. Available from: https://icer-review.org/wp-content/uploads/2018/07/ICER_SMA_Final_Evidence_Report_052419.pdf. (Accessed October 19, 2019).
6. Lally C, Jones C, Farwell W, et al: Indirect estimation of the prevalence of spinal muscular atrophy Type I, II, and III in the United States, *Orphanet J Rare Dis* 2017, 12: 175–180.
7. Kraszewski JN, Kay DM, Stevens CF, et al.: Pilot study of population-based newborn screening for spinal muscular atrophy in New York state, *Genet Med*. 2018, 20: 608–613.
8. Chien YH, Chiang SC, Weng WC, et al.: Presymptomatic diagnosis of spinal muscular atrophy through newborn screening. *J Pediatr.* 2017, 190: 124–129.
9. Vill K, Kölbel H, Schwartz O, et al.: One year of newborn screening for SMA - Results of a German pilot project. *J Neuromuscul Dis*. 2019, 6: 503–515.
10. Calucho M, Bernal S, Alías L, et al.: Correlation between SMA type and SMN2 copy number revisited: An analysis of 625 unrelated Spanish patients and a compilation of 2834 reported cases. *Neuromuscul Disord*. 2018, 28: 208–215.
11. RED BOOK, 2019. Available from: www.ibm.com/products/micromedex-red-book. (Accessed December 8, 2019).
12. Centers for Medicare and Medicaid Services (CMS) Physician Fee Schedule Search, 2018. Available from: https://www.cms.gov/apps/physician-fee-schedule/search/search-criteria.aspx. (Accessed November 2, 2018).
13. RED BOOK, 2018. Available from: www.ibm.com/products/micromedex-red-book. (Accessed November 2, 2018).

### P26

#### Co-production of the Welsh Rare Disease Research Gateway

##### Emma L. Hughes^1,2^, Rhian R. Morgan^1^ Angela M. Burgess^1,^

###### ^1^Wales Gene Park, Cardiff, Wales; ^2^Genetic Alliance UK, London, UK

**Correspondence:** Emma L. Hughes

*Orphanet Journal of Rare Diseases* 2020, **15(Suppl 1)**:P26

Patient and public involvement (PPI) is a key aspect of Wales Gene Park’s programme, particularly across education and engagement and prioritisation and development of research. In addition to representation on governance structures, Wales Gene Park (WGP) collaborates with patients and the public to involve them in rare disease and genetic research.

WGP has co-produced a Rare Disease Research Gateway following consultation with patients and the public from its networks. The Gateway hosts relevant studies in genetic and rare disease research on the WGP website. It promotes involvement opportunities in addition to signposting to studies that patients and other members of the public can participate in. It also links to training opportunities for PPI representatives.

Consultation with patients and the public regarding the usability, design and development of the Gateway was undertaken. Feedback has enhanced the user experience and it was launched in October 2019. There are currently over 230 studies featured, and the Gateway is searchable according to condition or key word. Impact will be monitored through online usage and website analytics.

Engagement with researchers through a Professional Network enables opportunities to be advertised from all areas of genetic and rare disease research and ensures that patient and public representatives are involved in the design and development of research from its inception. WGP were invited to present at the Welsh Health and Care Research Wales conference in 2019 as the Gateway was highlighted as an exemplar of good practice.

### P27

#### Economic burden of care and treatment options for patients with Rett syndrome: Two systematic literature reviews

##### Omar Dabbous^1^, Vanessa Taieb^2^, Emna Abdennadher^3^, Meryem Bouchemi^3^, Justyna Chorąży^4^, Katarzyna Borkowska^4^, Veneta Georgieva^5^, Bryan E. McGill^1^, Thomas A. Macek^1^, Benit Maru^1^, Ramesh Arjunji^1^

###### ^1^AveXis, Inc., Bannockburn, IL, United States; ^2^Creativ-Ceutical, London, United Kingdom; ^3^Creativ-Ceutical, Tunis, Tunisia; ^4^Creativ-Ceutical, Kraków, Poland; ^5^Creativ-Ceutical, Sofia, Bulgaria

**Correspondence:** Omar Dabbous

*Orphanet Journal of Rare Diseases* 2020, **15(Suppl 1)**:P27

**Background:** Rett syndrome, a rare X-linked disorder caused by *MECP2* mutations [1], occurs predominantly in girls and causes severe developmental impairment [2]. It is characterized by apparently normal development for the first 6–18 months of life, followed by the loss of acquired fine and gross motor skills and the ability to engage in social interaction, and the development of stereotypic hand movements [3]. Treatment options remain limited to targeting symptoms, leaving a significant unmet need [4]. Here, we assess the economic burden of care imposed on patients with Rett syndrome and their families and clinical trials on therapeutic approaches for Rett syndrome.

**Materials and methods:** Two systematic literature reviews related to Rett syndrome were performed in June 2018: (1) Related to economic burden (Medline, Embase, Cochrane Library, and Database of Abstracts of Reviews and Effects); (2) Clinical trials (Medline, Embase, ClinicalTrials.gov, and Cochrane Library). Outcomes of interest for economic burden were costs (direct and indirect medical costs), medical resource use (hospital admissions, length of stay, physician and specialist visit, medications) and non-medical resource use (lost productivity and homecare or caregiver’s time). Outcomes of interest for treatment options assessed the efficacy and safety of treatments for Rett syndrome.

**Results:** The search on economic burden yielded 133 articles; intervention type and costs were extracted from 9, representing 4 studies. In the economic burden studies, enteral feeding and assisted walking increased the risk of respiratory-related hospital admissions, while length-of-stay was lower in younger patients. Mean recovery-stay after scoliosis-correcting surgery was 18.2 days and 12.3 days in each of 2 studies. Care integration improved outcomes and reduced costs. The search on clinical trials yielded 652 articles; efficacy and safety were extracted from 28, representing 20 studies (15 randomized controlled trials, 5 single-arm; N = 8–82; follow-up 1–26 months). Of these, 19 focused on pharmacological symptom treatment; 1 examined environmental enrichment effects; none targeted the underlying cause. The most common primary endpoints are stated in Table 1. Naltrexone, trofinetide, and mecasermin demonstrated clinical benefits versus placebo, but most treatments yielded no significant improvement (Table 1).

**Table 1:** Statistically significant results from clinical studies

Outcome measure	Author, year	Intervention	Results (P-value)
RSBQ	Percy, et al. (2017) [5]	TFT 200 mg/kg vs PLC	Clinical benefit was observed for patients treated by TFT (0.042^T^)
CGI	Percy, et al. (2017) [5]	TFT 200 mg/kg vs PLC	Clinical benefit was observed for patients treated by TFT (0.029^T^)
VAS	Percy, et al. (2017) [5]	TFT 200 mg/kg vs PLC	Clinical benefit was observed for patients treated by TFT (0.025^T^)
	O’Leary, et al. (2018) [6]	PLC-MCS vs MCS-PLC	Worsening of symptoms for patients treated by PLC-MCS (0.0211^T^; 0.0111^W^)
ADAMS	O’Leary, et al. (2018) [6]	PLC-MCS vs MCS-PLC	Worsening of symptoms for patients treated by PLC-MCS (0.5535^T^; 0.0272^W^)
EEG	O’Leary, et al. (2018) [6]	PLC-MCS vs MCS-PLC	Worsening of symptoms for patients treated by PLC-MCS (0.0208^T^; 0.0110^W^)
	Gorbachevskaya, et al. (2001) [7]	CL vs UT	Clinical benefit was observed for patients treated with CL: lower value of LRP in alpha and beta bands (< 0.001^T^; < 0.01^T^), higher levels of LRP in the theta band (< 0.001^T^)
	Gorbachevskaya, et al. (2001) [7]	Before CL vs after CL	Improvement of the brain functional stage after treatment with CL: decrease of theta LRP in central and frontal regions (< 0.05^T^; < 0.01^T^), increase of beta activity LRP in the parietal region (< 0.05^T^), restoration of occipital alpha rhythm (< 0.05^T^)
ECG	Guideri, et al. (2005) [8]	ALC at BS vs ALC after 6 months	Clinical benefit was observed in patients treated with ALC: increase of total power (0.01^T^), VLF (0.01^T^), and LF (0.009^T^)
	Guideri, et al. (2005) [8]	UT at BS vs UT after 6 months	Decrease in heart rate variability was observed in UT: decrease of total power (0.04^T^) and LF (0.05^T^), and increase of QTcD (0.01^T^)
Respiratory function	Percy, et al. (1994) [9]	NLT vs PLC	Positive effect of NLT was observed: higher awake min. O2 saturation value (0.03^T^), less % time spent with disorganized breathing (0.02^T^), higher end tidal carbon dioxide value (0.02^T^)
	Djukic, et al. (2016) [10]	Before glatiramer acetate 20 mg vs after	Improvement of respiratory function: decrease of breath hold index (0.004^T^; 0.03^W^) and breath hold time (0.007^T^; 0.004^W^)
	Khwaja, et al. (2014) [11]	Pre MAD MCS vs post OLE	Improvement of respiratory function: improvement of apnea (0.012^T^)
CSS	Maffei, et al. (2014) [12]	ω-3 PUFAs at BS vs ω-3 PUFAs after 6 months	Significant improvements were observed: decrease in score for CSS (< 0.005^A^), ambulation (0.02^A^), hand use (0.002^A^), motor (0.009^A^), non-verbal communication (0.002^A^), and respiratory dysfunction (< 0.0001^A^)

A, Analysis of variance; ADAMS, Anxiety, Depression, and Mood Scale; ALC, acetyl-L-carnitine; BS, baseline; CGI, Clinical Global Impression; CL, cerebrolysin; CSS, Clinical Severity Scale; ECG, electrocardiogram; EEG, electroencephalogram; LF, low-frequency component (range: 0.04–0.15 Hz); LRP, logarithm of relative spectral power; MAD, multiple ascending dose; MCS, mecasermin; MCS-PLC, mecasermin for the first treatment period, placebo for the second; NLT, naltrexone; OLE, open-label extension; PLC, placebo; PLC-MCS, placebo for the first treatment period, mecasermin for the second; PUFA, polyunsaturated fatty acids; QTcD, QTc dispersion (difference between the min. and max. heart rate-adjusted QT interval among the 12 ECG leads); RSBQ, Rett Syndrome Behavioural Questionnaire; T, student’s t-test; TFT, trofinetide; UT, untreated; VAS, visual analog scale; VLF, very low-frequency component (< 0.04 Hz); W, Wilcoxon signed-rank test

**Conclusions:** Economic burden data were available for direct medical resource use and direct medical costs; data on non-medical and indirect resource use and costs within the last 5 years were not identified. Further research is needed to better understand the impact of medical interventions for patients with Rett syndrome and their potential to reduce costs and/or increase utility.

**References**

1. United Kingdom National Health Service. Available from: https:/www.nhs.uk/conditions/rett-syndrome. (Accessed: 30 March 2020).
2. International Rett Syndrome Foundation. Available from: https://www.rettsyndrome.org/about-rett-syndrome. (Accessed: 30 March 2020).
3. Orphanet. Available from: https://www.orpha.net/consor/cgi-bin/OC_Exp.php?Lng=GB&Expert=778. (Accessed: 30 March 2020).
4. AveXis. Press release, 17 June 2017. Available from: https://globenewswire.com (Accessed: 30 March 2020).
5. Percy AK, et al. Presented at AACAP’s 64th annual meeting. October 23–28, 2017.
6. O’Leary HM, Kaufmann WE, Barnes KV, et al.: Placebo- controlled crossover assessment of mecasermin for the treatment of Rett syndrome, *Ann Clin Transl Neurol*. 2018, 5: 323–32.
7. Gorbachevskaya N, Bashina V, Gratchev V, Iznak A: Cerebrolysin therapy in Rett syndrome: Clinical and EEG mapping study, *Brain Dev*. 2001, 23: S90–3.
8. Guideri F, Acampa M, Hayek Y, Zappella M: Effects of acetyl-L-carnitine on Cardiac Dysautonomia in Rett Syndrome: Prevention of Sudden Death?*,Pediatr Cardiol*. 2005, 26: 574–7.
9. Percy AK, Glaze DG, Schultz RJ, et al.: Rett syndrome: controlled study of an oral opiate antagonist, naltrexone, *Ann Neurol*. 1994, 35: 464–470.
10. Djukic A, Holtzer R, Shinnar Shlomo, et al.: Pharmacologic Treatment of Rett Syndrome With Glatiramer Acetate, *Pediatr Neurol*. 2016, 61: 51–57.
11. Khwaja OS, Ho E, Barnes KV, et al.: Safety, pharmacokinetics, and preliminary assessment of efficacy of mecasermin (recombinant human IGF-1) for the treatment of Rett syndrome, *Proc Natl Acad Sci USA.* 2014, 111: 4596–601.
12. Maffei S, De Felice C, Cannarile P, et al.: Effects of ω-3 PUFAs supplementation on myocardial function and oxidative stress markers in typical Rett syndrome, *Mediators Inflamm*. 2014, 983178.

### P28

#### CML Advocates Network implementation of the Community Advisory Board (CAB) model

##### Denis Costello^1^, Celia Marín^1^, Jan Geissler^1,2^, Pat García-González^1,3^

###### ^1^CML Advocates Network, Bern, Switzerland; ^2^Leukämie-Online LeukaNET, Bern, Switzerland; ^3^The Max Foundation, Seattle, WA, USA

**Correspondence:** Denis Costello - denis@cmladvocates.net

*Orphanet Journal of Rare Diseases* 2020, **15(Suppl 1)**:P28

The CML Advocates Network (CML AN) is an active network specifically for leaders of Chronic Myeloid Leukemia (CML) patient groups, connecting 123 patient organisations in 93 countries on all continents. It was set-up and is run by CML patients and carers. Its aim is to facilitate and support best practice sharing among patient advocates across the world. The CML Community Advisory Board (CML-CAB) is a working group of the CML Advocates Network. Since its inception the CML-CAB has met on nineteen occasions with five sponsors.

**Figure:**
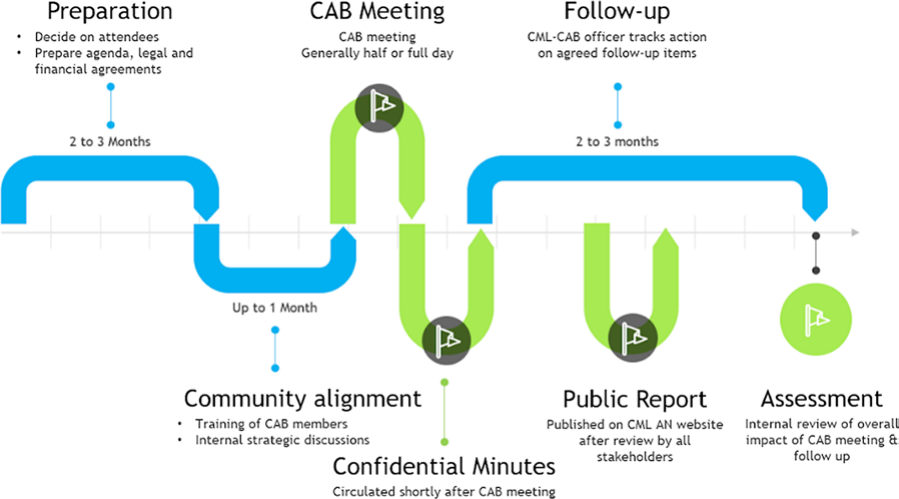
**Fig. 1** Outlines the process by which the CML-CAB operates

The CML-CAB is comprised of two chairs and 17 CAB-members. CML-CAB organisation, sustainability and follow-up is supported by a part-time CML-CAB Officer and the CML-AN Executive Director.

**Table 2:** The tools used by CML-AN as an internal management and measurement framework

Tool	Description
Protocol	This document defines the strategic objectives of the CML-CAB as well as defining roles and responsibilities for each CAB participant.
Confidentiality agreement	Addresses the scope of confidentiality discussions during CAB meetings and serves as a legal agreement
Follow-up Tracker	Allows CML-CAB and sponsor to mutually nominate responsibility for follow-up action
Documentation	Confidential record of the meeting plus a non-confidential public report for CML-AN members
Score card	Mechanism whereby CML-CAB can score the performance of the sponsor
Skills Matrix	Evaluation of the skills of CML-CAB members under the following domains: Research, Access, Collaboration, Drug Expertise and English language skills

The CML-CAB has discussed drug development pipelines, addressed access issues to treatments & monitoring, addressed collaboration issues, contributed to design of company-led patient services, and trained CML-CAB members.

## Speaker Presentations

### S1

#### Health determinants for universal health coverage in rare diseases

##### Vytenis P. Andriukaitis^1^, Birutė Tumienė^2,3^, Gediminas Černiauskas^4^

###### ^1^Former European Commissioner for Health and Food Safety, WHO Special Envoy for the European Region, Vilnius, Lithuania; ^2^Institute of Biomedical Sciences, Faculty of Medicine, Vilnius University, Vilnius, Lithuania; ^3^Vilnius University Hospital Santaros Klinikos, Vilnius, Lithuania; ^4^Health Economics Centre, Vilnius, Lithuania

*Orphanet Journal of Rare Diseases* 2020, **15(Suppl 1)**:S1

The principles of leaving no one behind are essential to the goals of World Health Organization (WHO) and United Nations (UN). In 2018, an ambitious objective to ensure that 1 billion more people will benefit from universal health coverage (UHC) until 2023 was entrenched in the WHO 13th General Programme of Work [1]. All UN Member States have agreed to try to achieve universal health coverage by 2030, as part of the Sustainable Development Goals [2]. However, it is essential, that rare disease (RD) patients are not left behind on our trip to UHC. In 2019, UN declared that RD are among the most vulnerable groups that are still on the fringes of UHC [3].

The first step on a way to the full UHC Cube [4] for RD is an identification of root causes of health inequities. Health determinants of RD fundamentally differ from those for common diseases. Some of them are unavoidable: up to 80% of RD have a genetic basis (individual or genetic determinants). Although socio-economic factors are highly important, in contrast to common diseases, they are a consequence rather than a cause of RD. Meanwhile, one of the major root cause amenable to change are health system determinants: organization of services for RD requires unique solutions in our health systems that are mostly adapted for common diseases. Political and legal determinants also play a key role: while RD is an explicit example of an area, loaded with needs for pan-European solutions, relative “weakness” of EU legal powers to regulate and have an impact on implementation of pan-European policies in health results in vast inequities among and inside Member States and lack of engagement at a national level. Health activism that includes strong advocacy and a loud voice of patient organizations has also been ascribed to health determinants and may have a crucial role in RD [5]

To improve the situation, we already have some powerful tools at hand including national plans for RD, European Reference Networks [6] and European Joint Programme on Rare Diseases [7]. However, to reach the full potential of these, multiple obstacles have to be removed and full implementation ensured.

**References**

1. World Health Organization. Thirteenth General Programme of Work 2019–2023. Promote Health - Keep the World Safe - Serve the Vulnerable. Available from URL: https://apps.who.int/iris/bitstream/handle/10665/324775/WHO-PRP-18.1-eng.pdf. Date accessed: 17 June 2020
2. UN General Assembly, Transforming our world: the 2030 Agenda for Sustainable Development, 21 October 2015, A/RES/70/1. Available at: https://www.refworld.org/docid/57b6e3e44.html. Date accessed: 17 June 2020
3. Political Declaration of the High-level Meeting on Universal Health Coverage “Universal health coverage: moving together to build a healthier world”, Sep 2019. Available at: https://www.un.org/pga/73/wp-content/uploads/sites/53/2019/07/FINAL-draft-UHC-Political-Declaration.pdf. Date accessed: 17 June 2020
4. World Health Organization. Making fair choices on the path to universal health coverage. Final report of the WHO Consultative Group on Equity and Universal Health Coverage. © World Health Organization 2014. Available at: https://apps.who.int/iris/bitstream/handle/10665/112671/9789241507158_eng.pdf?sequence=1. Date accessed: 17 June 2020
5. Friedman, E.A., Gostin, L.O. From local adaptation to activism and global solidarity: framing a research and innovation agenda towards true health equity. Int J Equity Health 16, 18 (2017). 10.1186/s12939-016-0492-8. Date accessed: 17 June 2020
6. European Commission website. European Reference Networks. Available at: https://ec.europa.eu/health/ern_en. Date accessed: 17 June 2020
7. European Joint Programme on Rare Diseases, Horizon 2020 research and innovation programme under grant agreement N°825575, https://www.ejprarediseases.org/. Date accessed: 17 June 2020

### S2

#### How digital health can support COVID-19

##### Liz Ashall-Payne

###### ORCHA, Daresbury, Warrington, UK, WA4 4AB

**Correspondence:** Liz Ashall-Payne - liz@orcha.co.uk

*Orphanet Journal of Rare Diseases* 2020, **15(Suppl 1)**:S2

Since March 2020, there has been an explosion in digital health adoption as people look for remote ways to manage their health and wellbeing. National Government COVID-19 strategies, local authorities and consumers, have all turned to health apps, both as a potential means of slowing the spread of the virus, and a method of allowing people to self-manage their own health.

In the first few weeks of the COVID-19 pandemic, ORCHA worked with app developers to build a dedicated COVID-19 App Library full of evaluated apps. Free to use for all, it included relevant, quality assured apps that had been through ORCHA’s rigorous Review process. To build such a tool in such a short space of time is testament to the speed of this market.

More consumers have been using health and care apps. In just one week, ORCHA saw an increase of 182.5% in app downloads from its App Libraries, and a 6,500% increase in app recommendations from health and care professionals.

ORCHA can see from the data across its App Libraries that the most popular search terms since the pandemic began have included: mental health, physiotherapy, fitness, anxiety, rehabilitation, diabetes, respiratory, and sleep.

Whereas ‘COVID’ was initially the most searched term at the beginning of the outbreak, people have since searched for specific condition areas. This indicates a shift in focus to actively self-managing health and wellbeing, and a desire for knowledge about particular health areas.

The recent increase in digital health adoption has highlighted that the challenge remains of helping consumers to understand which apps are potentially unsafe to use, and ensuring that consumers are armed with the full facts about the strengths and weaknesses of an app, before it is downloaded.

### S3

#### Expanded New born Screening – The Italian situation

##### Simona Bellagambi^1^, Annalisa Scopinaro^1^, Manuela Vaccarotto^2^

###### ^1^UNIAMOFIMR Federazione Italiana Malattie Rare, Rome, Italy; ^2^AISMME, Associazione Malattie Metaboliche Ereditarie, Padova, Italy

*Orphanet Journal of Rare Diseases* 2020, **15(Suppl 1)**:S3

In Italy Law167/2016 provided for the expansion of the neonatal screening to 38 metabolic diseases, in addition to phenylketonuria, congenital hypothyroidism and cystic fibrosis already screened since 1992 and the establishment of a National Coordination Centre(NCC) for Monitoring and Evaluation of Screening Centres and the inclusion of Expanded New Born Screening (ENS) in the Essential Level of Care providing the test for free for families and mandatory for the Regions. The NCC has the task of monitoring the uniform implementation in all Regions, provide the technical-scientific support to the health professionals involved and the creation of an archive of affected infants. The subsequent Ministerial Decree, was essential for the funding of the expanded test. Two further amendments have included further groups of diseases such as lysosomal diseases, primary immunodeficiencies and neuromuscular diseases and reduced the time for the update of the official panel. Additional funds were allocated to test the specific diseases identified within these groups thus added in the official panel. Their identification is postponed due to the pandemic. At the end of 2019, 19 out 20 Regions have fully implemented the ENS and experimental tests on further diseases are being carried out in some Regions for which the informed consent is needed. The working group for the identification of the specific diseases to be included in the panel and the elaboration of the pathway of the follow up, has not been established yet.

The achievement of the ENS was the result of the collaboration among the Metabolic Patient Organisations, UNIAMO Italian Federation of RDs, metabolic scientific society, public institutions and politicians. To cover and debate all aspects of the ENS system, the Italian Patient Organisations, gathered by UNIAMO, drafted a Position Paper with 10 Recommendations.

### S4

#### EPTRI - European Paediatric Translational Research Infrastructure and rare diseases

##### Donato Bonifazi

###### Consorzio per Valutazioni Biologiche e Farmacologiche, Pavia, Italy

**Correspondence:** Donato Bonifazi

*Orphanet Journal of Rare Diseases* 2020, **15(Suppl 1)**:S4

While considerable progresses have been made in the last years in research on innovative medicinal products for adults, children have not benefited from progresses to the same extent as adults in terms of appropriate treatments and advanced tools. It is well known that the availability of drugs for paediatric use still represents a challenging issue, since research and development in this field is characterized by many that range from methodological, ethical and economic reasons, especially when neonates and rare diseases are involved. Moreover, even when Industry has the capacity to perform a paediatric drug development plan, there are many economic reasons limiting the commercial sponsors’ interest (the paediatric population is a small population; paediatric diseases often concern rare disorders with unknown mechanism; it is very difficult to perform preclinical and clinical studies; ethical concerns are still relevant and additional regulatory requirements have to be considered).

In this scenario, EPTRI can make the different in closing the gap between innovative technologies and paediatric drug development processes. It is a EU-funded project that arises from the need to find answers to the serious lack of medicines for children in EU and worldwide, and aimed to design the framework for a European Paediatric Translational Research Infrastructure dedicated to paediatric research. An high interest is tailored on rare diseases (RD) as they affect mainly children and genetic RD start early in the prenatal/childhood life with an high frequent use of medicines not specifically tested (off-label, unlicensed). EPTRI will work to accelerate the paediatric drug development processes from medicines discovery, biomarkers identification and preclinical research to developmental pharmacology, age tailored formulations and medical devices. This will allow is to facilitate the translation of the acquired new knowledge and scientific innovation into paediatric clinical studies phases and medical use.

### S5

#### International perspectives on neonatal screening

##### Martina C. Cornel

###### Department of Clinical Genetics, AmsterdamUMC, Amsterdam, The Netherlands

**Correspondence:** Martina C. Cornel

*Orphanet Journal of Rare Diseases* 2020, **15(Suppl 1)**:S5

Neonatal screening started in many countries around 1960–1970 after phenylketonuria turned out to be a treatable condition. If diagnosed early, a diet could help to avoid impaired brain development. Public health programmes were developed to offer all newborn children the possibility to be tested. Screening always has benefits and disadvantages, and only rarely pros outweigh cons at reasonable costs. The World Health Organization in 1968 published criteria to evaluate benefits and disadvantages, concerning amongst others (1) important health problem (2) treatment (3) suitable test and (4) appropriate use of resources. PKU was mentioned as an example of an important health problem [1].

Neonatal screening is more than a test. Information to parents, communication of results, ICT infrastructure, follow-up of affected infants, reimbursement of test and treatment and governance all need adequate attention [2]. Around 2010 the number of diseases covered in European countries in neonatal screening programs was very diverse: from zero in Albania to more than 20 in Austria, Hungary, Iceland, Portugal and Spain [3]. Many countries have seen an increase in the number of diseases covered because of new tests and treatments becoming available. Health authorities were almost always involved in changes in the programmes, HTA experts and parents organizations sometimes. Half of the countries had laws on NBD, and half had a body overseeing NBS programs. Less than half of the countries informed parents of the storage of dried blood spots [3]. After the EU initiated “Tender NBS” had provided advice to EU policy makers [4], little initiatives for harmonization were taken, because health is the mandate of Member States. From the perspective of newborns this implies that early diagnosis and adequate treatment for NBS conditions may differ very much for children being born in one or another EU country. With more tests and more treatments becoming available, this makes it even more urgent to attune the perspectives of different EU stakeholders for the benefit of all newborns.

**References**

1. Wilson, J. and Jungner, G., 1968. Principles and Practice of Screening for Disease. [online] Apps.who.int. Available at: <https://apps.who.int/iris/handle/10665/37650> [Accessed 1 May 2020].
2. Burgard, P., Rupp, K., Lindner, M., Haege, G., Rigter, T., Weinreich, S., Loeber, J., Taruscio, D., Vittozzi, L., Cornel, M. and Hoffmann, G., 2012. Newborn screening programmes in Europe; arguments and efforts regarding harmonization. Part 2 – From screening laboratory results to treatment, follow-up and quality assurance. Journal of Inherited Metabolic Disease, 35(4), pp. 613–625.
3. Loeber, J., Burgard, P., Cornel, M., Rigter, T., Weinreich, S., Rupp, K., Hoffmann, G. and Vittozzi, L., 2012. Newborn screening programmes in Europe; arguments and efforts regarding harmonization. Part 1 - From blood spot to screening result. Journal of Inherited Metabolic Disease, 35(4), pp. 603–611.
4. Cornel, M., Rigter, T., Weinreich, S., Burgard, P., Hoffmann, G., Lindner, M., Gerard Loeber, J., Rupp, K., Taruscio, D. and Vittozzi, L., 2014. A framework to start the debate on neonatal screening policies in the EU: an Expert Opinion Document. European Journal of Human Genetics, 22(1), pp. 12–17.

### S6

#### What next after the search for a diagnosis? Hearing families’ experiences

##### Alessia Costa^1^, Vera Frankova^2^

###### ^1^Wellcome Genome Campus Society and Ethics Research, Wellcome Genome Campus, Hinxton, Cambridgeshire, CB10 1SA, UK; King’s College London, Faculty of Nursery, Midwifery & Palliative Care, SE1 8WA, UK; ^2^ Institute for Medical Humanities, First Faculty of Medicine, Charles University, 128 02 Prague 2, Czech Republic; Department of Biology and Medical Genetics, Second Medical Faculty, Charles University and Motol University Hospital, 150 06 Prague 5, Czech Republic

**Correspondence:** Alessia Costa - Alessia.costa@wgc.org.uk

*Orphanet Journal of Rare Diseases* 2020, **15(Suppl 1)**:S6

**Background**: As genome sequencing is rapidly moving from research to clinical practice, evidence is needed to understand the experience of patients with rare diseases and their families. In the presentation, we discuss families’ experience of receiving, making sense of and living with genomic information. The presentation includes video-clips from two short films from families’ narratives.

**Materials and methods**: We draw on filmed, narratives interviews with 17 families in the United Kingdom and Czech Republic. The interviews were coded and thematically analysed.

**Results**: we present findings on three qualitative themes: (i) families’ experience of receiving results; (ii) what happens next; (iii) the personal utility of genomic testing from families’ perspective.

**Conclusions**: the findings indicate that a diagnosis is not the end of families’ journey. Communication about genomic testing was often focused on facilitating families understanding what the result ‘is’, and opportunities were needed to enable families to formulate and discuss questions about what it ‘meant’ to them. Moreover, families often continued to experience significant uncertainty in their everyday life, particularly in the case of new, ultra-rare diagnoses. Specifically, families struggled with the lack of information on the course of the disease, the difficulties to access support and navigate health and social care services, and the challenges related to making sense of the implications of genomic information for other family members. Despite these issues, families identified a wide range of benefits from taking part in genome sequencing, which were broader than the clinical utility of the diagnosis. The findings raise questions regarding how to talk about ‘diagnosis’ in a way that reflects families’ experience, including their uncertainty but also their perceived benefits. They also have implications for the design and delivery of health services in the genomic era, pointing to the need to better support families after their search for a diagnosis.

**Acknowledgements**: We thank all the families who took part in the interviews, the staff of the health services and charities who collaborated to advertise the study to eligible participants and the members of the family advisory groups who reviewed the interview schedule and provided invaluable feedback on the preliminary findings.

The work has been presented on behalf of the study “Improving the Communication of Genomic Diagnosis Results Using Experience Based Co-Design (EBCD)”, which is part of the Solve RD project. The Solve-RD project has received funding from the European Union’s Horizon 2020 research and innovation programme under grant agreement No 779257.

### S7

#### STRIMVELIS® case study

##### Michela Gabaldo^1,2^, Maddalena Migliavacca^2,3^, Federica Barzaghi^2,3^, Maria Pia Cicalese^2,3^, Alessandro Aiuti^2,3,4^

###### ^1^Fondazione Telethon, Milan, Italy; ^2^San Raffaele Telethon Institute for Gene Therapy, San Raffaele Scientific Insitute, Milan, Italy; ^3^Pediatric Immunohematology and Bone Marrow Transplantation Unit, San Raffaele Scientific Institute, Milan, Italy; ^4^Vita-Salute San Raffaele University, Milan, Italy

*Orphanet Journal of Rare Diseases* 2020, **15(Suppl 1)**:S7

**Background:** Strimvelis is the first ex-vivo autologous stem/progenitor cell (HSPC) gene therapy (GT) registered in 2016 in Europe for the treatment of the ultra-rare ADA-SCID patients. In the absence of an HLA-identical sibling, allogeneic transplantation from alternative donors is affected by a significant degree of mortality (30–50%). GT has been developed as academic product up to the clinical proof of concept and then licensed to a pharma company in 2010 who completed the regulatory steps enabling the registration and market access. The commercial product is available in a single center in Milan.

**Case report:** The product is a successful case study where different stakeholders (a charity and an academic hospital through the SR-TIGET, San Raffaele Telethon Institute for gene therapy) joint their complementary efforts to research, develop and successfully translate into the clinics this innovative product while the ATMP Regulation entered into force. In 2010 SR-TIGET established the 1st strategic alliance with a pharma company to complement competencies and financial effort to complete the industrialization, prepare the registrational dossier and access the market. The product is fresh and it is available to any eligible patient at a single center in Milan, Italy.

The 1st patient has been treated in 2000 and is doing well. Since then, 36 patients from 19 countries have been treated, 12 of whom with the commercial product. All patients are alive and the safety and efficacy profile seen during the development has been confirmed with experience administering the commercial product, with no evidence of insertional mutagenesis. Monitoring of patients treated with Strimvelis is ongoing in the context of a long-term prospective registry study.

**Conclusion:** Strimvelis is a successful case study of a product developed in academia and then partnered with pharma to make it finally available to any patient in need as a commercial product.

### S8

#### SALUSCOOP - new social institution to management of data for common good

##### Joan Guanyabens

###### SALUSCOOP, Barcelona, Spain

**Correspondence:** Joan Guanyabens - joan@guanyabens.com

*Orphanet Journal of Rare Diseases* 2020, **15(Suppl 1)**:S8

Saluscoop [http://www.saluscoop.org] is a non-profit data cooperative for health research that aims to make a greater amount and diversity of data available to a broader set of health researchers, and to help citizens to manage their data for the common good.

Data heals. Health research is data-driven: the larger the universe, the greater the quantity, quality, and diversity of the data, the more potential the data has to cure.

In our European context, it is clear: data belongs citizen. GDPR regulates ownership and our rights over data that include portability. Data protection laws rightly consider that health data deserves the maximum protection. However, the only truth, we note every day: In practice citizen often cannot access their data or control its use.

The future of our health depends significantly on the ability to combine, integrate and share personal health data from different sources.

The only one who can integrate all your information (public, private, clinical, personal, habits, genetics) is the citizen himself. Using data well, it is possible to obtain More and Better health for all.

We are a Cooperative that works to facilitate the transformation process towards this goals doing:

- Dissemination, awareness, communication – Studies, Manifestos.
- Licenses to facilitate it - SALUS Common Good license
- Instruments - App Salus

It is necessary to dissociate the provision of services, of the possession of the data. The accumulation and centralization of the data is not necessary. Blockchain and the like allow the certification of transactions without the need for intermediaries.

The need for the existence of new social institutions for the collective management of data for the common good is much clearer today:

- So that these citizens have the technological and legal tools effectively manage their data.
- So that health research can address the real problems of our societies.

**Acknowledgements:** The abstract is being presented on behalf of a SALUSCOOP Management Board Group.

### S9

#### Integrated plan and case management for holistic care in rare diseases: innovative experience from the region of Murcia-Spain

##### Encarna Guillén-Navarro^1^, Juan Carrión-Tudela^2^, Joaquín A. Palomar-Rodríguez^3^, M. Teresa Martínez-Ros^3^

###### ^1^Sección de Genética Médica. Hospital Clínico Universitario Virgen de la Arrixaca, Instituto Murciano de Investigación Sanitaria (IMIB-Arrixaca), Universidad de Murcia (UMU), Murcia, Spain. Asociación Española de Genética Humana (AEGH), Spain; ^2^Federación Española de Enfermedades Raras (FEDER), Madrid, Spain; ^3^Consejería de Salud de la Región de Murcia, Spain

**Correspondence:** Encarna Guillén-Navarro - eguillen@um.es; guillen.encarna@gmail.com

*Orphanet Journal of Rare Diseases* 2020, **15(Suppl 1)**:S9

**Background:** Rare Diseases (RD) have high complexity, chronicity and impact on the life expectancy and quality. An important barrier for holistic care in Spain is the absence of Clinical Genetics as health specialty, since most of RD are genetics.

The Region of Murcia, located on the southeast of Spain, has 1.5 million inhabitants. In 2015, approximately 5% of its population was identified with a RD, based on the Regional RD Information System, which showed a public health problem requiring an integral and coordinated approach.

**Results:** In 2018, after 2 years of participative work (interdepartmental government representatives, patients associations and professionals) the Regional Plan for RD integrated (holistic) care was approved, for a period of 4 years (2018–2021) and a budget of 12 millions euros; with the goal of improving health, education and social care through interdisciplinary coordination and placing patients and families in the center of the actions. The plan includes ten different strategic areas related to information, prevention and early detection, healthcare, therapeutic resources, social-health care, social services, education, training of professionals, research, monitoring and evaluation

A Regional RD Coordination Center, linked to the Medical Genetics Unit in the tertiary reference hospital, is connected to the 9 health areas, educational and social local services, through a case manager integrated in the multidisciplinary team. This was our building experience presented in the INNOVCare project, co-funded by the EU.

**Conclusions:** To design a holistic care plan for RD we need to know the prevalence based on RD registries, available and needed resources and an interdisciplinary participative action approach with the appropriate government and financial support with periodic evaluation. Case management has an important role. The recognition of Clinical Genetics as health specialty is also urgent in Spain to provide equal access to RD patients and families all over the country.

### S10

#### “Live longer, healthier lives”: Rare Disease Population Needs 2030 (and beyond)

##### Victoria Hedley^1^, Dalia Aminoff^2^, Kate Bushby^3^, Matt Bolz-Johnson^4^

###### ^1^Newcastle University John Walton Muscular Dystrophy Research Centre, Institute of Genetic Medicine, Newcastle University, Newcastle, UK; ^2^A.I.M.A.R, Rome, Italy; ^3^Institute of Genetic Medicine, International Centre for Life, Newcastle upon Tyne, UK; ^4^EURORDIS – Rare Diseases Europe, Koeln, Germany

**Correspondence:** Victoria Hedley - victoria.hedley@ncl.ac.uk

*Orphanet Journal of Rare Diseases* 2020, **15(Suppl 1)**:S10

Looking back over the past decade or so, the European RD field can celebrate several major achievements, which were made possible by ‘soft law’ such as the 2008 Commission Communication [1] and 2009 Council Recommendation [2] - and legislation such as Directive 2011/24 EU [3]. These policies have served us well, but it is essential that the policies guiding us towards the future we wish to see are equipped to address the needs of the future RD population. The Rare 2030 project [4] is working towards precisely this goal, and has identified over a hundred future-facing trends likely to impact on the field.

Some of these trends concern demographic changes about which we can be reasonably certain: whilst overwhelmingly positive, changes such as ageing RD populations will bring new challenges in managing comorbidities. They will also create new opportunities as well as risks in areas such as reproductive choice; however, these choices incur major ethical, legal and social concerns, and it is unclear how many countries really have robust frameworks in place to cope with this.

Besides the fairly certain demographic changes, there are many topics -and many needs- for which the future is not clear. Will there be easier access to expert multidisciplinary teams? What will be the role of technology in care delivery? These fundamental issues are here debated in interview format [5].

**References**

1. Communication from the Commission to the European Parliament, the Council, the European Economic and Social Committee and the Committee of the Regions on Rare Diseases: Europe’s challenges [Internet]. Ec.europa.eu. 2008 [Accessed 15 May 2020]. Available from: https://ec.europa.eu/health/ph_threats/non_com/docs/rare_com_en.pdf
2. Council recommendation of 8 June 2009 on an action in the field of rare diseases (2009/C 151/02). [Internet]. eur-lex. europa.eu. 2009 [Accessed 18 September 2015]. Available from: http://eur-lex.europa.eu/LexUriServ/LexUriServ.do?uri OJ:C:2009:151:0007:0010:EN:PDF
3. Directive 2011/24/EU of the European Parliament and of the Council of 9 March 2011 on the application of patients’ rights in cross-border healthcare [Internet]. Eurlex.europa.eu. 2011 [Accessed 9 March 2015]. Available from: http://eurlex.europa.eu/LexUriServ/LexUriServ.do?uri=OJ:L:2011:088:0045:0065:en:PDF
4. Rare2030 [Internet]. Rare2030.eu. 2020 [Accessed 15 May 2020]. Available from: https://www.rare2030.eu/
5. “Live longer, healthier lives”: Rare Disease Population Needs 2030 (and beyond) [Internet]. 2020 [cited 18 May 2020]. Available from: https://drive.google.com/file/d/11SFE6xp09deIsCOGrbwSWHT15UZnXERJ/view?usp=sharing

### S11

#### A case study of Adrenoleukodystrophy (ALD): Alex The Leukodystrophy Charity’s application to the UK National Screening Committee

##### Sara Hunt

###### Alex, The Leukodystrophy Charity, London, SE15 5 EB, UK

**Correspondence:** Sara Hunt

*Orphanet Journal of Rare Diseases* 2020, **15(Suppl 1)**:S11

Adrenoleukodystrophy, or ALD, is a complex x-linked genetic brain disorder which mainly affects males between the ages of four and 60 – males who are previously perfectly healthy and ‘normal’. ALD damages the myelin in the brain and spinal cord, and those with cerebral symptoms become completely dependent on their loved ones or carers. This usually involves patients becoming wheelchair or bed bound, blind, unable to speak or communicate and tube fed. It is a difficult disorder to diagnose with behaviour problems usually the primary indicator.

In males, cerebral ALD is a terminal illness with most dying within one to 10 years of symptoms developing. If diagnosed before symptoms become apparent, usually through identification of a family member, the condition can be successfully treated through bone marrow transplant.

Some adults (males and females) develop a related condition called adrenomyeloneuropathy, or AMN. Symptoms include difficulty walking, bladder and bowel incontinence and sexual dysfunction. Tragically, around one third of males with AMN go on to develop cerebral ALD. Initial behavioural symptoms often have an impact on the individual’s professional and personal lives – their capacity to work, maintain relationships and family ties – over time, they can become isolated and socially unacceptable. Commonly, those individuals without supportive family structures are missed or misdiagnosed.

The presentation presents a personal case study detailing the impact of an ALD diagnosis on the whole family, moving on to Alex TLC’s experience in applying to add ALD to the UK’s New Born Screening Programme. The conclusion includes next steps following an initial negative response, and thoughts on the methods used to assess decisions on the prevention and treatment of rare disease.

### S12

#### GestaltMatch: Identifying Facial Phenotypes of Genetic Disorders Using Deep Learning

##### Tzung-Chien Hsieh^1^, Aviram Bar-Haim^2^, Guy Nadav^2^, Jean Tori Pantel^1,3^, Nicole Fleischer^2^, Peter Krawitz^1^

###### ^1^Institute of Genomic Statistics and Bioinformatics, University of Bonn, Bonn, Germany; ^2^FDNA Inc., Boston Massachusetts, MA 02111, United States; ^3^Charité – Universitätsmedizin Berlin, corporate member of Freie Universität Berlin, Humboldt-Universität zu Berlin, and Berlin Institute of Health, Institute of Medical Genetics and Human Genetics, Berlin, Germany

**Correspondence:** Peter Krawitz - pkrawitz@uni-bonn.de

*Orphanet Journal of Rare Diseases* 2020, **15(Suppl 1)**:S12

**Introduction:** Recent advances in next-generation phenotyping (NGP) for syndromology, such as DeepGestalt, have learned phenotype representations of multiple disorders by training on thousands of patient photos. However, many Mendelian syndromes are still not represented by existing NGP tools, as only a handful of patients were diagnosed. Moreover, the current architecture for syndrome classification, e.g., in DeepGestalt, is trained “end-to-end”, that is photos of molecularly confirmed cases are presented to the network and a node in the output layer, that will correspond to this syndrome, is maximized in its activity during training. This approach will not be applicable to any syndrome that was not part of the training set, and it cannot explain similarities among patients. Therefore, we propose “GestaltMatch” as an extension of DeepGestalt that utilizes the similarities among patients to identify syndromic patients by their facial gestalt to extend the coverage of NGP tools.

**Methods:** We compiled a dataset consisting of 21,400 patients with 1,451 different rare disorders. For each individual, a frontal photo and the molecularly confirmed diagnosis were available. We considered the deep convolutional neural network (DCNN) in DeepGestalt as a composition of a feature encoder and a classifier. The last fully-connected layer in the feature encoder was taken as Facial Phenotypic Descriptor (FPD). We trained the DCNN on the patients’ frontal photos to optimize the FPD and to define a Clinical Face Phenotype Space (CFPS). The similarities among each patient were quantified by cosine distance in CFPS.

**Results:** Patients with similar syndromic phenotypes were located in close proximity in the CFPS. Ranking syndromes by distance in CFPS, we first showed that GestaltMatch provides a better generalization of syndromic features than a face recognition model that was only trained on healthy individuals. Moreover, we achieved 87% top-10 accuracy in identifying rare Mendelian diseases that were excluded from the training set. We further proved that the distinguishability of syndromic disorders does not correlate with its prevalence.

**Conclusions:** GestaltMatch enables matching novel phenotypes and thus complements related molecular approaches.

### S13

#### Rare 2030 Foresight Study: Overview and presentation of four Rare 2030 “What If” scenarios

##### Milan Macek^1^, Anna Kole^2^

###### ^1^Rare 2030 Research Advisory Board Member, Professor of Medical and Molecular Genetics, Motol University Hospital and Charles University Prague, Czech Republic; ^2^EURORDIS - Rare Diseases Europe, Paris, France

**Correspondence:** Anna Kole - anna.kole@eurordis.org

*Orphanet Journal of Rare Diseases* 2020, **15(Suppl 1)**:S13

The Rare2030 foresight study gathers the input of a large group key opinion leaders through an iterative process to propose recommendations for a new policy framework for people living with rare diseases (RD) in Europe.

Since the adoption of the Council Recommendation on European Action in the field of RD in 2009, the European Union has fostered tremendous progress in improving the lives of people living with RD. Rare2030 will recommendations for the next ten years and beyond.

The Rare2030 Foresight Study includes 4 major stages (Fig. 1).

**Figure:**
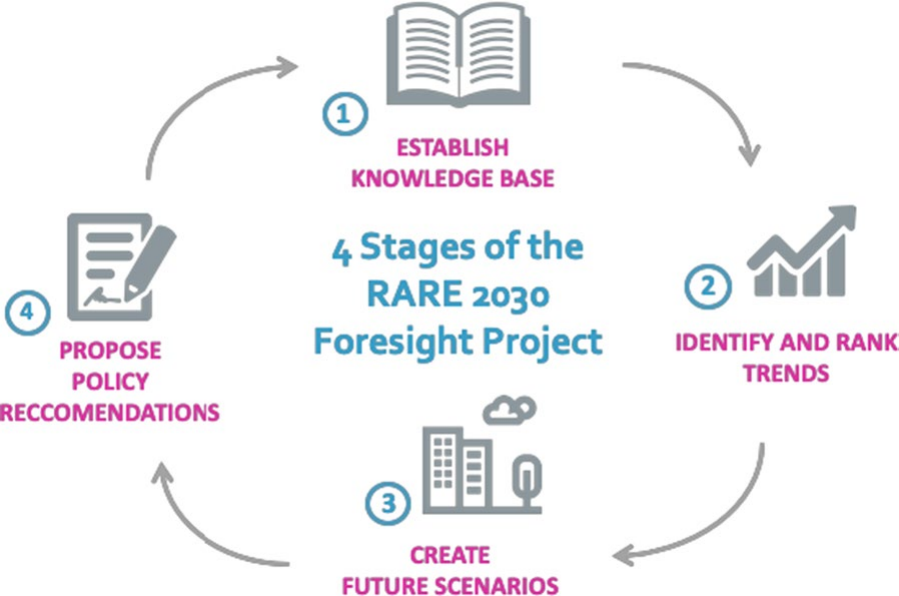
**Fig. 1** Four stages of the Rare 2030 Foresight process include (1) Establishing a knowledge base (literature review), (2) Identifying and Ranking Trends (horizon scanning), (3) Creating Scenarios, (4) Proposing Policy Recommendations

The European Conference on Rare Diseases and Orphan Products (ECRD 2020) marked the occasion to present four proposed future scenarios (Fig. 2, Video 1) prepared through literature review and horizon scanning by an 8-partner project consortium and 200-member panel of experts.

**Figure:**
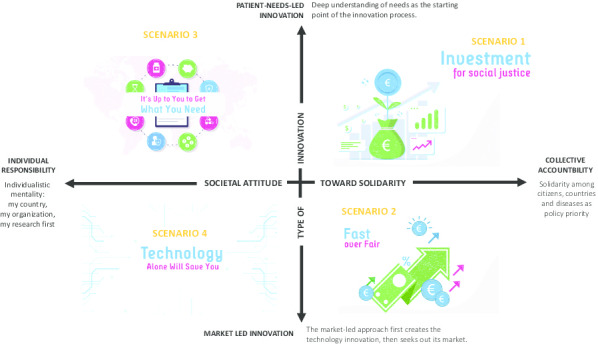
**Fig. 2** Four possible scenarios for people living with a rare disease were created along two axes by combining different progressions of key trends – (horizontal) trends related to societal attitudes towards solidarity and (vertical) trends related to drivers of innovation

The conference launched discussions around the following questions:

1. Which scenarios are most preferred by the RD community?
2. Which scenarios are most likely to happen?
3. How do we achieve the scenarios we prefer and avoid those we don’t? What are the policies needed to do this?

An audience of over 800 delegates voted on the Rare2030 scenarios and discussions throughout the sessions of ECRD 2020 indicated the following opinions:

- If we continue as we are we will find ourselves in the “Fast over Fair” scenario which forecasts high collective responsibility but an emphasis on market-led innovation
- The majority of the audience preferred a future scenario with continued high collective accountability but more of an emphasis on needs-led innovation, “Investments for Social Justice”
- A significant portion of the audience agreed that a balance must remain with the market led attractiveness of the “Technology Along Will Save You” scenario
- A scenario where “It’s Up to You to Get What You Need” was least preferred by all

**Acknowledgements:** The Rare2030 Consortium is led by EURORDIS-Rare Diseases Europe tasked with the strategic/scientific coordination of the project. ISINNOVA is responsible for the administrative and operative management and all guidance on foresight methods. Six additional partners – Orphanet, Newcastle University’s Institute of Genetic Medicine, Fondazione Telethon, European Reference Network for Hereditary Metabolic Disorders (University Hospital of Udine), European Reference Network on Rare Bone Disorders (Istituto Ortopedico Rizzoli), Imperial College of London Centre for Health Economics and Policy Innovation complete the Executive Management Committee in executing the project’s objectives. A multi-stakeholder Panel of Experts comprised of over 200 thought leaders in the field of rare diseases and health serve as the central consultative body during the Foresight study.

### S14

#### Reducing the Diagnostic Odyssey Through Medical Education

##### Lucy McKay

###### Medics4RareDiseases, Gloucestershire, UK

**Correspondence:** Lucy McKay - lucy@m4rd.org

*Orphanet Journal of Rare Diseases* 2020, **15(Suppl 1)**:S14

The diagnostic pathway in rare disease has a number of bottlenecks that can result in the pathway becoming an odyssey. While some barriers are being removed through remarkable innovation, there is one story of diagnostic delay that is echoed by rare disease patients across the globe and across thousands of different rare diseases: doctors failed to suspect something rare.

However we cannot expect doctors to suspect rare diseases when they haven’t been trained to or, in some cases, have been trained to do the exact opposite with the mantra “common things are common”. Without appropriate training ‘rare’ can be mistaken for ‘irrelevant’ when in reality 30 million European citizens live with a rare disease [1].

Medics4RareDiseases is driving an attitude change towards rare diseases in the medical profession. This begins with explaining that rare diseases are collectively common and all clinicians should expect to manage people with diagnosed and undiagnosed rare disease regularly during their careers. This attitude change is called #daretothinkrare.

Secondly M4RD is suggesting a new approach to educating about rare disease for trainers and training institutes. This approach tackles rare disease as a collective and focuses on patient needs rather than details of individual diseases. This not only solves the impossible challenge of covering over 7000 rare diseases during medical training but also provides some equity between different diseases.

Lastly, M4RD promotes the use of rare disease specific resources that will support both doctors and their patients. This includes the invaluable input from patient advocacy groups. The step between presenting with symptoms and being suspected of having a rare disease can be the longest in many steps to getting a diagnosis. This is something we have the power to change now by providing content tailored to medics, early in their careers that will equip them to #daretothinkrare.

**Reference**

1. About Rare Diseases | www.eurordis.org [Internet]. Eurordis.org. 2020 [Accessed 4 June 2020]. Available from: https://www.eurordis.org/about-rare-diseases

### S15

#### New disruptive technologies: How can we prepare healthcare systems? An industry view

##### Francis Pang

###### Orchard Therapeutics, London, UK

**Correspondence:** Francis Pang

*Orphanet Journal of Rare Diseases* 2020, **15(Suppl 1)**:S15

To prepare for delivery of gene therapies, companies typically focus on four key areas: patient identification & diagnostics; treatment centre qualification; manufacturing & supply and market access. Timely diagnosis of patients is important as with progressive disorders, the earlier patients are treated, typically the better their long-term clinical outcomes will be. Targeted tools and resources are used to educate clinical specialists on the early symptoms of the disease. Improving access to the appropriate diagnostic tests is essential. If newborn screening is considered, validated assays and pilot studies are required. Gene therapies have to be administered in qualified treatment centres. After regulatory approval, treatment centres are relatively few so patients may need to cross borders and work is required to expand the recognition of patient rights to be treated in another EU country (e.g. through the S2 mechanism). Many companies partner with contract manufacturing organisations and are developing ways to preserve gene-corrected stem cells to enable their transportation from the manufacturing site to treatment centres. The final area is market access, whereby it is vital to evolve the way healthcare systems think about delivery, funding and value determination. Manufacturers have the responsibility to generate health economic evidence. Recent research [1] in metachromatic leukodystrophy showed that caregivers (n = 17) spend an average of 17 hours a day caring for their child. 83% of parents were forced to miss work with 68% of this being unpaid leave. In addition, it is recommended to have the optionality of payment models that allow the sharing of risk between the healthcare system and manufacturer (e.g. annuity or outcomes-based payments). Orchard has developed a holistic value framework as gene therapies are expected to benefit patients, families, communities, healthcare systems and society

**Reference**

1. Walz M, Calcagni C, Wilds A, Howie K, Shapovalov Y, Pang F. Caregiver-reported impact on quality of life and disease burden in patients diagnosed with Metachromatic Leukodystrophy: Results of an online survey and a qualitative interview. 16th Annual WORLD Symposium 2020

### S16

#### Rare and equal in job employment – my daily self-management practice and my involvement in patients’ organisations

##### Jana Stefanova Popova

###### EAMDA/ EPF Youth Group; Ljubljana, Republic of Slovenia; Brussels, Belgium;

**Correspondence:** Jana Stefanova Popova - jana.s.popova@gmail.com

*Orphanet Journal of Rare Diseases* 2020, **15(Suppl 1)**:S16

**Background:** Employment has always been one of the fundamental human rights. It is important for people with rare diseases, because it helps to stay connected to the community and to continue professional development. Equal access to job employment can help to overcome the consequences of the condition and to gain financial independence. On the other hand unemployment can increase the social exclusion.

**Materials and methods:** In the last few years there is an improvement in the European policies about job employment. In spite of this, people with rare diseases still have to overcome discrimination in this field. As a proof of this statement is the recent online survey, conducted by EURORDIS. According to it, 70% of the respondents admitted they had to reduce their professional activities after they were diagnosed with rare condition. This means that more than half of the people with rare diseases in Europe face employment challenges. The analysis of this survey was important input to the presentation of the EPF Youth Group project – WAYS.

**Results:** This is the abbreviation of Work and Youth Strategy and it is a two year project, disseminated among young patients with chronic conditions. The main purpose was to increase the awareness about positive and negative practices for young patients on the labour market and to develop recommendations to employers and decision makers. That is why EPF Youth Group conducted an online survey and provided different deliverables like factsheet with recommendations to employers and video about young patients’ rights on the work place.

**Conclusion:** The results of both survey provided important insight about the challenges people with rare diseases face in job employment. It proved the fact that only if we work together as a community of patients, we will be able to provide better opportunities for national and international inclusion.

### S17

#### Reshaping Patient Access with Decentralized Registries

##### Paul Rieger^1^, Eberhard Scheuer^2^

###### ^1^Centiva Health AG, Zug, Switzerland

**Correspondence:** Paul Rieger - paul@centiva.health

*Orphanet Journal of Rare Diseases* 2020, **15(Suppl 1)**:S17

The lack of access to research participants is the number one reason why medical studies fail [1]. Real-world data is often difficult to get despite USD 70 billion costs of patient data intermediation. Therefore, a new model for patient access is necessary where patients get paid fairly for their data, retain control over their data, and drive citizen-centered research. On the other hand, researchers and industry must be enabled to access patients directly without violating their privacy, while reducing time and costs of data access at the same time.

Current patient registries facilitate patient access and match patients with a centralized data flow while giving little to no incentives. Whereas, a decentralized patient registry allows for direct and confidential matchmaking between patients and organizations looking for data through the use of blockchain technology. It lets the patient decide with whom they want to share their data. On such a platform, patients can receive incentives in the form of digital currency. Currently, Centiva Health [2] is used in the context of rare diseases and population health, i.e., outbreak monitoring.

In the area of rare diseases Centiva Health cooperates with patient advocacy groups by enhancing existing registries with the ability to collect real-world data. The access to patient via a decentralized registry leads to aligned incentives, real-time access to data, improved disease visibility while preserving patient privacy.

**References**

1. Fogel DB. Factors associated with clinical trials that fail and opportunities for improving the likelihood of success: A review. Contemp Clin Trials Commun. 2018;11:156–164. Published 2018 Aug 7. 10.1016/j.conctc.2018.08.001
2. https://www.centiva.health/ [retrieved, 5 June 2020]

### S18

#### Improving the Communication of Genomic Diagnosis Results Using Experience Based Co-Design (EBCD)

##### Glenn Robert

###### King’s College London, Faculty of Nursery, Midwifery & Palliative Care, SE1 8WA, UK

**Correspondence:** Glenn Robert

*Orphanet Journal of Rare Diseases* 2020, **15(Suppl 1)**:S18

**Background:** To help inform cross-national development of genomic care pathways, we worked with families of patients with rare diseases and health professionals from two European genetic services, one in the United Kingdom and in the Czech Republic, to co-design optimal methods/services for the communication of genomic results.

**Methods and results:** Using a methodology called Experience-Based Co-Design (EBCD)^1^, we supported families and health professionals to shared and discuss their experiences, identify priories for improvement and then work together to prototype and test out interventions to address these. The process involved observations of clinical appointments (), interviews with families () and health professionals and a series of workshops and remote consultations at both sites.

**Results:** Five shared priorities for improvement were identified by participants at the two sites, and eight quality improvement interventions were prototyped/tested to address these (Table 1).

**Discussion**: the findings clearly indicate the need for improved follow-up care to support families in the short, medium term after the sharing of the results, including when a diagnosis is confirmed. Different service models were prototyped, including follow up consultations with clinical geneticists and a dedicated role to facilitate co-ordinated care. The findings also demonstrate the need for continued workforce development on the psychosocial aspects of genomic and genetic communication, specifically on families’ needs regarding genomic consent and the experience of guilt and (self-)blame.

**Table 1:** Study outcomes at both sites

Priorities for improvement		Quality improvement interventions
*UK Site*		
a. Communication at the point of testing		1. Principles and recommendation to improve communication with families 2. Use of film for workforce engagement and professional development (on-going)
b. Named person for follow up and questions		3. Job description: Liaison Officer for rare/undiagnosed diseases
*Czech Site*		
a. Follow-up care		1. Follow up appointment within 1 month
b. Prevention of feeling of guilt		2. Workshop on families’ experience of guilt and (self-)blame 3. Educational resources at the Department 4. Referral pathways for families in need of specialist psychosocial support (on-going)
c. Environment of the Department		5. Environmental improvements (on-going)

**Acknowledgements**

We thank all the families who took part in the interviews, the staff of the health services and charities who collaborated to advertise the study to eligible participants and the members of the family advisory groups who reviewed the interview schedule and provided invaluable feedback on the preliminary findings.

The work has been presented on behalf of the study “Improving the Communication of Genomic Diagnosis Results Using Experience Based Co-Design (EBCD)”, which is part of the Solve RD project. The Solve-RD project has received funding from the European Union’s Horizon 2020 research and innovation programme under grant agreement No 779257.

**Reference**

1. Bate P, Robert G. Bringing user experience to health care improvement: the concepts, methods and practices of experience-based design. Oxford: Radcliffe Publishing; 2007

### S19

#### From rare data to FAIR data for rare diseases

##### Marco Roos^1^, Mark D. Wilkinson^2^, Ronald Cornet^3^, Deborah Mascalzoni^4^, Veronica Popa^5^, Ian Harrow^6^,Claudio Carta^7^, Yaffa R. Rubinstein^8^, Dipak Kalra^9^, Ana Rath^10^, Victoria Hedley^11^, Gülçin Gümüş^12^

###### ^1^Human Genetics Department, Leiden University Medical Centre, Leiden, The Netherlands; ^2^Departamento de Biotecnología-Biología Vegetal, Escuela Técnica Superior de Ingeniería Agronómica, Alimentaria y de Biosistemas, Centro de Biotecnología y Genómica de Plantas, Universidad Politécnica de Madrid (UPM) - Instituto Nacional de Investigación y Tecnología Agraria y Alimentaria (INIA), Madrid, Madrid, Spain; ^3^Department of Medical Informatics, Amsterdam UMC, Amsterdam Public Health, Amsterdam, Netherlands; ^4^Institute for biomedicine, Eurac Research, Bolzano, Italy; ^5^MCT8-AHDS Foundation, Oklahoma, 74464, United States; ^6^Ian Harrow Consulting, Whitstable, UK; ^7^Istituto Superiore di Sanità, Rome, Italy; ^8^Special volunteer at the National Institutes of Health, NLM, Bethesda, MD 20894, United States; ^9^The European Institute for Innovation through Health Data (i~HD), Gent, Belgium; ^10^Orphanet, Inserm-US14, Rare Diseases Platform, Paris, France; ^11^Newcastle University, Newcastle-upon-Tyne, UK; ^12^EURORDIS-Rare Diseases Europe, Barcelona, Spain

**Correspondence:** info-rdsgofair@go-fair.org (Marco Roos - m.roos@lumc.nl, Gülçin Gümüş - gulcin.gumus@eurordis.org)

*Orphanet Journal of Rare Diseases* 2020, **15(Suppl 1)**:S19

Rare disease data are a critical resource for researchers, clinicians, and patients. Because data are sparse, it is necessary to enable analysis across organisations and countries. Concrete examples of multi-source questions are “What ages are associated with ambulation loss due to steroid use in muscular dystrophies?”, “How does treatment of severe nosebleeds compare between expert centres?”, “What study can I contribute to as a patient?”. In theory, multi-source analysis, from simple questions to advanced AI, can often be performed in seconds if data are compliant with FAIR data principles: Findable, Accessible, Interoperable, and Reusable for humans and computers [https://www.youtube.com/watch?v=_V8y0IedaqE]. In practice, it can take months of searching data, understanding the sources, mapping to consistent standards, and negotiating how one might use the data.

Many assume that for sharing and analysis, data need to be moved between sources. This can lead to sharing only minimal, non-sensitive data: a fraction of global rare disease data. Alternatively, data elements and local access conditions can be described by globally agreed, computer understandable standards conform FAIR principles. This enables analysis at each source, while sharing only the analysis results. FAIR prepares data for rapid discovery, access, and analysis, also when data remain at source. Projects such as the European Joint Programme for Rare Diseases work on the technical infrastructure to support this.

Adopting FAIR principles requires culture change. FAIR advocates working on rare diseases have organised the ‘Rare Diseases Global Open FAIR implementation network’ (RDs GO FAIR) to foster this change [https://www.go-fair.org/implementation-networks/overview/rare-diseases/]. RDs GO FAIR prioritizes patient representatives for their capacity to reshape current practices, welcoming them to organise their own network within RDs GO FAIR to foster FAIR for patient priorities (registration for follow-up meetings is possible via [https://tinyurl.com/y7mu2r9s])

**Acknowledgements:** We would like to thank all seed group members of the Rare Diseases Global Open FAIR Implementation Network, the GO FAIR Office, EURORDIS, the European Union’s Horizon 2020 research and innovation program under the EJP RD COFUND-EJP N° 825575, the RD-Connect community, the LUMC Biosemantics research group, Simone Louisse (GuardHeart ePAG), and the many patients and patient representatives that inspire us.

### S20

#### A framework of quality assurance of rare disease Centers of Excellence and European Reference Networks

##### Birute Tumiene^1^, Matt Bolz-Johnson^2^, Ines Hernando^3^, Alberto M. Pereira^4^, Anke Widenmann-Grolig^5^, Till Voigtländer^6^

###### ^1^Institute of Biomedical Sciences, Faculty of Medicine, Vilnius University, Lithuania; ^2^Healthcare and ERN Advisor, EURORDIS, Koeln, Germany; ^3^Healthcare and ERN Director, EURORDIS, Brussels, Belgium; ^4^ENDO-ERN Coordinator; Head of the Division of Endocrinology, Chair Centre for Endocrine Tumors Leiden (CETL), Leiden University Medical Centre, Leiden, The Netherlands; ^5^KEKS & EAT, Stuttgart, Germany; ^6^Medical University of Vienna, Vienna, Austria

*Orphanet Journal of Rare Diseases* 2020, **15(Suppl 1)**:S20

Quality assurance of rare disease (RD) Centers of Excellence (CoE) through designation, accreditation, monitoring and constant improvement provides a means to ensure high quality, centralization of resources and expertise, and cost-efficiency. EUCERD recommendations for quality criteria of CoE, issued in 2011, are still highly relevant [1]. In the State of Art Resource, almost all European Union (EU) Member States (MS) claim, that their CoEs conform to EUCERD recommendations [2]. However, national quality assurance processes differ significantly: some MS apply robust procedures, while in other MS, many of them - but not exclusively - are EU-13 MS, processes of quality assurance are less developed. Under the subsidiarity principle embedded into European treaties, the EU plays a limited role in many areas of healthcare, and CoEs quality assurance processes are a choice and responsibility of MS.

With the establishment of ERNs, another layer of quality assurance has been developed by the European Commission and the MS [3]. This new quality assurance framework may be in line, or not, with national accreditation systems and involves i) assessment of CoEs when they apply for Full Membership of ERNs and ii) continuous monitoring afterwards [4]. In every ERN, Members have to be “equal partners in the game” and share the same goals, rights and obligations. While the ERN logo should eventually be a quality mark of the highest standards, strong links of ERN Members to national systems, including many more and less specialized healthcare providers, are essential to ensure proper care pathways for RD patients.

Importantly, ERNs themselves and patients/non-governmental organizations provide us with additional means of “informal quality assurance”. Many ERNs are implementing their own monitoring processes through the creation of registries to collect health outcomes that allow peer-benchmarking. Meanwhile, patients provide their strong voice through European Patient Advocacy Groups (ePAGs) and help to signpost “the best” CoEs through information sharing. In both these processes, the power of open, transparent information on performance may finally lead to improved transparency and accountability at a national level and, presumably, may have an impact on the composition of ERNs in the future.

**References**

1. EUCERD Recommendations on Quality Criteria for Centres of Expertise for Rare Diseases in Member States, 24 October 2011. [Accessed 17 June 2020] Available at: http://www.EUCERD.eu/upload/file/EUCERDRecommendationCE.pdf.
2. Joint Action RD-ACTION (European Union’s Health Programme 2014–2020, No.677024), State of the Art Resource, available at: http://www.rd-action.eu/rare-disease-policies-in-europe/. [Accessed 17 June 2020]
3. European Commission website. [Accessed 17 June 2020]. European Reference Networks. Board of Member States, available at: https://ec.europa.eu/health/ern/board_member_states_en.
4. [Internet]. Ec.europa.eu. 2020 [Accessed 17 June 2020]. Available from: https://ec.europa.eu/health/sites/health/files/ern/docs/continuous_monitoring_en.pdf

### S21

#### Patient’s view on disruptive innovations in clinical research

##### Elizabeth Vroom^1,2^

###### ^1^Duchenne Parent Project, Amsterdam, The Netherlands; ^2^World Duchenne Organization, Veenendaal, The Netherlands

**Correspondence:** Elizabeth Vroom - elizabeth.vroom@worldduchenne.org

*Orphanet Journal of Rare Diseases* 2020, **15(Suppl 1)**:S21

In order to improve clinical research, patient preferences and outcome measures relevant to patients should become the core of drug development and be implemented from the earliest stage of drug development. From ‘bedside to bench’ instead of from ‘bench to bedside’.

At all levels the reuse of data could and should be enhanced. Patient derived or provided data are not owned by those who collected them, and their reuse should be primarily controlled by the donors of these data. Researchers and Health professionals are custodians (GDPR). To enable the optimal reuse of real world data, the data needs to be Findable, Accessible, Interoperable and Reusable (FAIR) by medical professionals, patients and in particular also by machines. For this reason the World Duchenne Organization published a Duchenne FAIR Data Declaration [1]. Reuse of placebo data and use of natural history data could speed up research especially in the field of Rare Diseases

At this moment, in line with GDPR, patients are in a good position to decide about the reuse of their own data and should not only have access to these data but preferable also be in charge of their own data. By collecting relevant data (Patient Reported Outcome Measures), storing their own clinical data from different data silo’s (Electronic Health Records (EHRs), registries and companies) and deciding themselves with whom to share. Duchenne Parent Project together with Foundation29 set up a Duchenne Data Platform that facilitates all mentioned before.

Also trial design could profit from the reuse of data. Input from patients in trial design in general should improve as well. Projects such as trials@home (IMI) may help to change the future of clinical trials and the use of Community Advisory Boards (EURORDIS) should be encouraged as much as possible.

**Reference**

1. FAIR Declaration - World Duchenne [Internet]. World Duchenne. 2020 [Accessed 5 June 2020]. Available from: https://www.worldduchenne.org/fair-declaration/

### S22

#### Repurposing technology to create a new normal for hidden disabilities

##### Allison J. Watson^1^, Tim Buckinx^2^

###### ^1^Ring20 Research and Support UK CIO, Brentwood, Essex, UK; ^2^Epihunter, Hasselt, Belgium

**Correspondence:** Allison J. Watson - allison@ring20researchsupport.co.uk

*Orphanet Journal of Rare Diseases* 2020, **15(Suppl 1)**:S22

**Background:** Drug repurposing for rare disease has brought more cost-effective and timely treatment options to patients compared to traditional orphan drug development, however this approach focuses purely on medical interventions and requires extensive clinical trials prior to approval. In the case of refractory epilepsy, practical solutions are also required to better manage daily life. Here we present an example of technology repurposing as a practical aid to managing absence epilepsy.

**Methodology:** Existing research tells us that seizure control is not the only consideration of Quality of Life in children with epilepsy and that mental health and caregiver/peer support are of utmost importance. We explored the needs of stakeholders and determined that there was a delicate balance between the individual (and those that care for them) and those that have the power to change their lives.

**Results:** Across all stakeholders there was a shared common need to obtain objective data on absence monitoring to relieve the burden on families/carers to retain manual seizure diaries whilst providing accurate and timely data to medical teams, researchers and social care.

Epihunter is an absence seizure tracking software using repurposed technology: a headset from wellness/leisure to collect electroencephalographic (EEG) data and an AI algorithm to detect and record absence seizures on a mobile phone application in real-time. Both EEG and video recording of the seizure are automatically captured. This low cost, easy to use technology has been used in the home to provide objective seizure data prior to upcoming clinic appointments. The COVID-19 pandemic has prompted an acceleration in telemedicine and epihunter has improved the effectiveness of virtual consultations bringing opportunities for both diagnostics and informed changes in treatment.

**Conclusions:** Epihunter is an example of technology repurposing to create a new normal for people with hidden disabilities such as those living with absence epilepsy.

### S23

#### Patients Perspective on ‘need for centralization of care’ and mobilizing system change

##### Anke Widenmann-Grolig

###### EAT e.V. The Federation of Esophageal Atresia and Tracheo-Esophageal Fistula Support Groups

**Correspondence:** Anke Widenmann-Grolig

*Orphanet Journal of Rare Diseases* 2020, **15(Suppl 1)**:S23

2013 in Germany the National Action Plan for Rare Diseases (NAMSE) has been published with a detailed description of criteria on three levels:

1. A-Centers include research and training activities, are an expert center for more than one diagnosis.
2. B-Centers fulfill all criteria for one diagnosis and are collaborating with an A-Center and other B-Centers for the same diagnosis.
3. C-Centers are working together with a B-Center for the local/regional care of the patients.

The rare disease patient community tried to get this well detailed plan to be transferred to regulation which usually means an adequate financial substitution of those expert services.

The patients should benefit from a centralized expert treatment/care pathway.

Esophageal atresia (EA) is a rare congenital condition with an estimated prevalence of 1 to 2 in 5,000 live births. Esophageal atresia patients require life-long attention.

ERNICA has developed a ‘patient journey’ for EA patients, under the leadership of patient representatives from the international federation of EA support groups (EAT).

**Figure:**
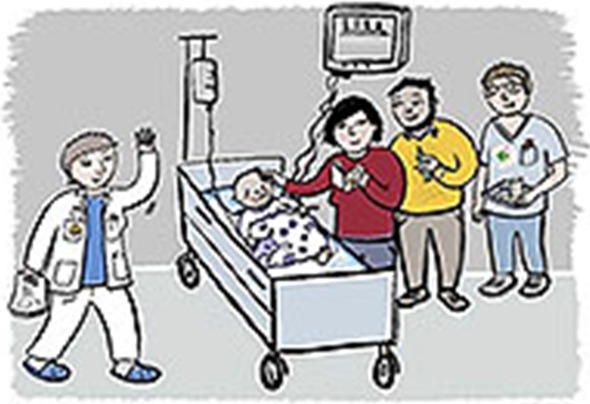
**Fig. 1** Example of patient journey stage depicted pictorially

In Germany, patients with congenital malformations which need surgery in early life are treated in hospitals with (very) low experience.

How can we as patient representatives get the fruits of the ERNs into the national health system?

We don’t have public money. We have no official contract and no political support.

KEKS e.V., the German EA support group together with other support groups (e.g. SoMA e.V.), and with surgical expert teams across Germany, some of them members in ERNs, started to organize monthly virtual boards for those patients.

A self-commitment on ethical and medical standards following the ERN-criteria, and a collaborative attitude within the group, help us to get step by step the first ERNICA results to the bedside of EA patients.

